# Targeting B7-H3 via chimeric antigen receptor T cells and bispecific killer cell engagers augments antitumor response of cytotoxic lymphocytes

**DOI:** 10.1186/s13045-020-01024-8

**Published:** 2021-01-29

**Authors:** Jie Liu, Shuo Yang, Bihui Cao, Guangyu Zhou, Fengjuan Zhang, Yuan Wang, Rixin Wang, Lipeng Zhu, Ya Meng, Cong Hu, Hui Liang, Xu Lin, Kangshun Zhu, Guokai Chen, Kathy Qian Luo, Lijun Di, Qi Zhao

**Affiliations:** 1 Cancer Centre, Faculty of Health Sciences, University of Macau, Taipa, Macau SPR China; 2 Institute of Translational Medicine, Faculty of Health Sciences, University of Macau, Taipa, Macau SPR China; 3 Department of Minimally Invasive Interventional Radiology and Department of Radiology, The Second Affiliated Hospital of Guangzhou Medical University, Guangzhou, Guangdong China; 4 Zhuhai People’s Hospital Affiliated with Jinan University, Zhuhai, Guangdong China

**Keywords:** B7-H3, Chimeric antigen receptor, Bispecific antibody, Non-small cell lung cancer, PD-L1, BiKE, CAR T

## Abstract

**Background:**

B7-H3, an immune-checkpoint molecule and a transmembrane protein, is overexpressed in non-small cell lung cancer (NSCLC), making it an attractive therapeutic target. Here, we aimed to systematically evaluate the value of B7-H3 as a target in NSCLC via T cells expressing B7-H3-specific chimeric antigen receptors (CARs) and bispecific killer cell engager (BiKE)-redirected natural killer (NK) cells.

**Methods:**

We generated B7-H3 CAR and B7-H3/CD16 BiKE derived from an anti-B7-H3 antibody omburtamab that has been shown to preferentially bind tumor tissues and has been safely used in humans in early-phase clinical trials. Antitumor efficacy and induced-immune response of CAR and BiKE were evaluated in vitro and in vivo. The effects of B7-H3 on aerobic glycolysis in NSCLC cells were further investigated.

**Results:**

B7-H3 CAR-T cells effectively inhibited NSCLC tumorigenesis in vitro and in vivo. B7-H3 redirection promoted highly specific T-cell infiltration into tumors. Additionally, NK cell activity could be specially triggered by B7-H3/CD16 BiKE through direct CD16 signaling, resulting in significant increase in NK cell activation and target cell death. BiKE improved antitumor efficacy mediated by NK cells in vitro and in vivo, regardless of the cell surface target antigen density on tumor tissues. Furthermore, we found that anti-B7-H3 blockade might alter tumor glucose metabolism via the reactive oxygen species-mediated pathway.

**Conclusions:**

Together, our results suggest that B7-H3 may serve as a target for NSCLC therapy and support the further development of two therapeutic agents in the preclinical and clinical studies.

## Background

Lung cancer is the most common type of cancer worldwide and is associated with the highest mortality among all common cancers. Non-small cell lung carcinoma (NSCLC) is a major subtype of epithelial lung cancer and accounts for a majority of cases [1]. Metastatic spread of cancer is the reason for most NSCLC-related deaths [2]. Lung cancer cells frequently metastasize to some major organs, such as bone, brain, lung and liver [3]. Although most NSCLC patients receive multiple treatments, including surgery, radiation, chemotherapy, targeted therapy, NSCLC still exhibits low cure rate, high recurrence and mortality [4]. Attempts have been made to develop new strategies for the treatment for NSCLC [5]. To date, immunotherapy has shown promise in patients with metastatic NSCLC.

In recent years, immune checkpoint therapy becomes a breakthrough strategy to reactivate antitumor immune responses [6]. Programmed cell death protein 1 (PD-1) and its ligand PD-L1 axis, as an inhibitory immune checkpoint signaling pathway, play a crucial role in the progression of tumor. In particular, inhibiting PD-1 and its ligand PD-L1 axis has shown remarkably results in NSCLC treatment. Currently, two antibodies (nivolumab and pembrolizumab) against PD-1 and two (atezolizumab and durvalumab) against PD-L1 have been approved for treating advanced stage NSCLC [7]. However, anti-PD-1/PD-L1 therapy remains challenging because of the low response rates [8]. Multiple factors, including T-cell infiltration, neoantigen burden and tumor metabolism, are involved in the immune checkpoint blockade [9].

B7-H3, also known as CD276, is a type I transmembrane protein that shares up to 30% amino acid identity with PD-L1 [10, 11]. The receptor of the B7-H3 protein remains unclear. B7-H3 contributes to a co-inhibitory immune signal during modulation of cytotoxic lymphocyte function in cancer immunity [12]. While B7-H3 protein is expressed at low levels in most normal tissues, it is aberrantly expressed on differentiated malignant cells and cancer-initiating cells, with limited heterogeneity, and in multiple tumor types, including lung, colon, breast and ovarian cancers [13]. In head and neck squamous cell cancer and pancreatic ductal adenocarcinoma, high B7-H3 expression is observed on cancer-initiating cells. Moreover, it is overexpressed in the tumor vasculature and stroma fibroblasts [14, 15]. B7-H3 plays an important role in tumor immune evasion and metastasis [16]. In NSCLC patients, B7-H3 overexpression is frequently associated with lower level of tumor-infiltrating lymphocytes [17]. B7-H3 expression﻿ may be correlated with EGFR gene expression status and efficacy of anti-PD-1 therapy [18]. Recent evidence indicated that B7-H3 expression potentially involves in resistance to anti-PD-1/PD-L1 blockade in NSCLC and ovarian cancer [18, 19]. B7-H3 represents an attractive target for antibody-based immunotherapy. However, the molecular mechanisms of B7-H3-modulation of cancer immunity are not clear.

﻿Due to its broad expression across a panel of tumor types, B7-H3 becomes an attractive therapeutic target. Recently, several monoclonal antibodies (mAbs) targeting B7-H3 have generated promising results against pancreatic adenocarcinoma [20], glioblastoma [21, 22], pediatric solid tumors [23, 24], and lymphoma [25]. Specifically, recently developed anti-B7-H3 mAb, 8H9 (omburtamab) and its humanized forms, inhibited the growth of different B7-H3-positive tumor cells through antibody-dependent cell-mediated cytotoxicity (ADCC) or as immunoconjugates in preclinical [26–29]. ^124^I could be safely delivered with 8H9 by direct injection into human pontine gliomas for both PET imaging and therapy [30], while ^131^I-8H9 administered to the cerebrospinal fluid showed potential in improving survival among patients with metastasis to the central nervous system and the leptomeninges [24].

Adoptive immunotherapy that utilizes effector lymphocytes expressing tumor-specific antibodies is a promising approach to treat cancer [31–33]. Genetic modifications using B7-H3 chimeric antigen receptors (CARs) gave promising results in xenografts of pediatric tumors [20, 23], glioblastoma [21] and melenoma [34]. To facilitate immune cell responses, two modalities, CARs and bispecific killer cell engagers (BiKE), use single chain variable fragments (scFvs) to redirect cytotoxic lymphocytes against specific antigens that are expressed on tumor cells [35]. These novel strategies have emerged as potentially curative therapies in the treatment for leukemia and some solid tumors. Here, we aimed to systematically evaluate the value of B7-H3 as a target in NSCLC via T cells expressing B7-H3-specific CARs and BiKE-redirected natural killer (NK) cells.

## Methods

### Human tissue samples

Either formalin-fixed paraffin-embedded or surgical tissue samples were obtained from the Second Affiliated Hospital, Guangzhou Medical University, and Zhuhai People’s Hospital, Jinan University. ﻿Buffy coat samples collected from healthy adult donors were obtained from Macau Blood Center. All procedures were in accordance with the ethical standards approved by the human ethics committees.

### RNA-sequencing analysis

The B7-H3 mRNA expression in 110 normal lung tissues, 488 lung adenocarcinoma (LUAD) samples and 509 lung squamous cell carcinoma (LUSC) samples with different stages was analyzed using R packages ggplot2 and ggbeeswarm. Data were downloaded from the Cancer Genome Atlas (TCGA) (http://www.oncolnc.org).

### Cell lines and cell culture conditions

The HEK293, Daudi, MDA-MB-231, NCI-H23, HCC827, A549, BT-474, OVCAR-3, SK-OV-3 cell lines were obtained from the Stem Cell Bank, Chinese Academy of Sciences. HCC 1954 cell line was obtained from MSKCC. DLD-1, HCT 116 and PANC-1 cell lines were provided by Prof. Hang-Fai Kwok. SF188 and U251 cell lines are provided by Prof Gang Li. OCI-AML-3 and MOLM-13 cell line was provided by Dr. Tong-Kam Leung. These cell lines were cultured in either RPMI-1640, DMEM or DMEM/F12 supplemented with 10% fetal bovine serum (FBS) (GIBCO),100 U/mL penicillin and 100 µg/mL streptomycin (GIBCO) at 37 °C with 5% CO_2_. The FreeStyle 293-F cells were obtained from Invitrogen and cultured in Freestyle 293 expression medium (GIBCO) in the incubator shaker set at 125 rpm, 8% CO_2_, and 37 °C.

### Antibodies

Antibodies for APC-CD3, PE-CD4, PE/Cy7-CD8, Alexa Fluor647-CD56, PE-CD107a, PE-CCR7, PE/Cy7-CD62L, PE/Cy7-perforin, PE-granzyme B, PE-PD-L1 were purchased from BioLegend. Goat anti-B7-H3 antibody (MAB1027) was purchased from R&D System. Humanized anti-B7-H3 IgG (8H9) was expressed and purified in the laboratory (for details, see Additional file 1). Mouse anti-CD16 antibody (3G8) was purchased from BD Biosciences. PE-conjugated anti-human Fc antibody and HRP anti-human IgG antibody were purchased from Invitrogen. HRP-conjugated rabbit anti-goat IgG antibodies were purchased from Jackson ImmunoResearch. Rabbit antihuman PD-L1 antibody (13,684), HRP-conjugated anti-β-actin and anti-GAPDH antibodies were purchased from Cell Signaling.

### Generation of B7-H3-specific CAR-T cells

The DNA sequence of the single chain variable fragment (scFv) of 8H9 antibody was synthesized and then introduced into the pLVX lentivirus backbone plasmid, containing E1α promoter, a CD8 leader sequence, CD8-α transmembrane domain, a 4-1BB and a CD3ζ intracellular signaling domains. Lentiviruses carrying the B7-H3-CAR were produced in HEK293 cells that were co-transfected with B7-H3-CAR lentiviral vector and packaging plasmids (PsPAX2 and pMD2.0G) as previously described [36]. The lentiviral supernatants were collected at 72 h after the transfection and filtrated through a 0.45 µm filter (Millipore), followed by centrifugation for 2 h at 28,000 rpm. For titer calculation, HEK293T cells (5 × 10^5^ per well) were seeded into 12-well plate and cultured to reach 80% confluence. The different dilutions of lentiviruses were mixed with polybrene at 5 μg/ml in 1 ml fresh medium. The culture medium in the wells was replaced by the mixture of lentiviruses and incubated for 2 days. After harvesting, the infection percentages of HEK293T cells were counted based on Zsgreeen detection by the flow cytometer. The positive ratio of each well was recorded. The titer can be calculated from cell counting using the following formula:

$${\text{titer}}\left( {\text{TU/ml}} \right) = \frac{{{\text{Total}}\;{\text{cell}}\;{\text{number }} \times {\text{positive}}\;{\text{rate}}}}{{{\text{added}}\;{\text{volume}}\;{\text{of }}\;{\text{virus}}\left( {{\mu l}} \right){ }}} \times 1000.$$

Human peripheral blood mononuclear cells (PBMCs) were isolated from buffy coats using Ficoll reagents (GE Healthcare) and cultured in complete RPMI-1640 supplemented with 2 mM L-glutamine, 100U/mL penicillin, 100 μg/mL streptomycin, 10% FBS and 100U/mL r-human IL-2 (rhIL-2) (CellGenix). PBMCs were stimulated by Dynabeads Human T-Activator CD3/CD28 (Gibco) at a 1:1 T cell: bead ratio in the growth medium supplemented with rhIL-2. One day after activation, the activated T cells were transduced with the lentivirus at a multiplicity of infection (MOI) of 5. Spin infection was performed with polybrene at 570 × g for 1 h at 32 °C, followed by incubation for 2 days. The anti-CD3/CD28 beads were magnetically removed on day 7. T cells were expanded in complete RPMI-1640 until used in vitro or in vivo assays*.* Cells were fed every 2 days and used within 20 days of expansion in all experiments. Vehicle control T cells were produced in the same conditions.

### Production of the B7-H3/CD16 BiKE

The scFv of the humanized anti-B7-H3 antibody 8H9 and the scFv of anti-CD16 antibody 3G8 were generated by coupling of heavy chain variable region (VH) and light chain variable region (VL) via the (GGGGS)3 linker, respectively. The scFvs were cloned into pComb3x vector. To generate a BiKE targeting B7-H3 and CD16, the anti-B7-H3 scFv 8H9 and anti-CD16 scFv 3G8 were linked with an additional (GGGGS)3 linker and then cloned into the pSecTag B expression vector. Freestyle 293-F cells were used to express bispecific antibodies. Transfection into HEK293 cells was performed as described previously [37]. The soluble scFv was expressed and purified as previously described [26]. The anti-B7-H3 x CD16 bsAb, anti-B7-H3 scFv and anti-CD16 scFv were purified using Ni–NTA agarose beads (Qiagen).

### Cytotoxicity assays

Cytotoxicity of CAR-T cells was measured using the Calcein-AM release method as previously described with modifications [38]. Targeted cells at 1 × 10^6^ cells/mL were incubated with 10 μM of Calcein-AM for 30 min at 37 °C. For CAR-T cells, targeted cells seeded at 1 × 10^4^ cells/well in the 96-well plate were co-incubated with effector CAR-T cells at different effector-to-target (E:T) ratios from 5:1 to 40:1 in a total volume of 200 μL for 4 h.

ADCC assay was performed using PBMC as effectors as previously described with modifications [39]. Targeted cells were labeled with Calcein-AM. Different concentrations of antibodies were incubated with the mixture of tumor cells and PBMC at a 10:1 E:T ratio in a total volume of 200 μL for 4 h. The spontaneous release control wells and maximum release target control wells were set up in all experiments. Mean fluorescence intensity (MFI) was measured using PerkinElmer Multimode Reader at 495/515 nm. The specific lysis ratios were calculated according to the formula:

$${\text{lysis}}\;{\text{ratio }}\left( \% \right) = \frac{{{\text{MFI }}\left( {{\text{sample }}\;{\text{lysis}}} \right) - {\text{MFI }}\left( {{\text{spontaneous}}\;{\text{ release}}} \right)}}{{{\text{MFI }}\left({{\text{maximum}} \;{\text{lysis}}} \right) - {\text{MFI }}\left( {{\text{spontaneous}}\;{\text{release}}} \right)}} \times 100\% .$$

### Xenografted mouse models

All animal studies were conducted using protocols approved by the Animal Ethics Committees, University of Macau. Six- to eight-week-old female NOD/SCID mice were bred in the Animal Facility at Faculty of Health Sciences. Six- to eight-week-old female NOD/SCID mice were randomly divided into groups (n = 5 per group). Each mouse was subcutaneously inoculated with 100 μL PBS containing 1.5 × 10^6^ tumor cells. To investigate the therapeutic effect of B7-H3-specific CAR-T cells, 5 days after tumor inoculation, mice in each group were intravenously treated with PBS, PBS containing 1 × 10^7^ CAR-T cells or vehicle T cells per mouse on day 0 and day 7, respectively. To investigate the therapeutic effect of anti-B7-H3 × CD16 BiKE, antibodies (BiKE, B7-H3 scFv, CD16 scFv, 100 μg per mouse) were administered intravenously twice a week for a total of four doses. In animals that also received human effector cells with or without antibodies, PBMCs from healthy donors were injected intravenously into mice (1 × 10^7^ cells per mouse). Tumor growth was measured using digital calipers twice a week, and tumor volumes were calculated using the formula *V* = ½(length × width^2^).

### Flow cytometry

Human B7-H3 expression on cell surface of tumor cells was detected using anti-B7-H3 IgG 8H9, followed by a fluorescently-labeled anti-human Fc antibody. For cytokine measurement of CAR-T cells, target cells (A549 and HCT 116) were seeded at 1 × 10^5^ cells/well in a 96-well plate and co-incubated with effector B7-H3-CAR T cells at an E:T ratio of 10:1 for 6 h or 24 h. Cytokine (IFN-γ, IL-2, IL-4, IL-6, IL-10 and TNF-α) secretion in the culture supernatants was measured using BD™ Cytometric Bead Array Human Th1/Th2 Cytokine Kit II (BD Biosciences) following the manufacture’s instruction. To determine T cell differentiation, CAR-T cells were co-incubated with tumor cells (A549 and HCT 116) in a E:T of 10:1 for 6–24 h, followed by antibody staining for CD3, CD4, CD8, CD107a, CCR7, CD62L, and apoptosis dyes (7-AAD and Annexin V). For measurement for intracellular perforin and granzyme B, T cells were fixed and permeabilized, followed by stained with an anti-perforin antibody and an anti-granzyme B antibody, respectively. All samples were analyzed using the CytoFLEX S (Beckman Coulter) or Accuri™ C6 (BD Biosciences) flow cytometry. Data were processed using FlowJo software.

### Dynamic apoptotic detection by FRET

A fluorescence resonance energy transfer (FRET)-based measurement of caspase-3 was used to determine the real-time apoptosis in tumor cells [40]. Briefly, A549 cells carrying caspase-3 biosensor (A549-C3) were seeded 1 × 10^5^ cells/well in the 24-well plate and incubated at 37 °C, 5% CO_2_ to grow until confluence. After 1 day, 1 × 10^6^ PBMCs with or without 5 μg/mL BiKE were added to the plate. Then, cells were incubated for different periods to observe the time-dependent FRET changes. The fluorescent images of living cells were acquired using a fluorescence microscope (Zeiss Axio Observer 7) with an excitation at 436 ± 10 nm, and the emission for YFP detection at 535 ± 12.5 nm and CFP at 480 ± 15 nm. Imaging data were analyzed using ZEN software (Zeiss).

### Immunohistochemistry

For immunohistochemistry (IHC), the samples were fixed with 10% formalin and processed for paraffin embedding. Sectioned slices were deparaffinized in xylene and rehydrated in graded alcohol, and placed in Tris-buffered saline (TBS) for 15 min. After the antigen retrieval and inactivation of endogenous peroxidase, sections were blocked with animal nonimmune serum (Maxvision) and incubated with goat anti-human B7-H3 primary antibodies MAB1027 and 8H9 overnight at 4 °C, respectively. After the incubation with corresponding secondary antibodies (Maxvision) for 15 min, the sections were stained using the detection kit (Maxvision). Cell nuclei were stained with hematoxylin (Sigma). Finally, sections were dehydrated with absolute ethanol. The pictures of samples were captured in the Olympus TH4-200 microscope.

### ECAR and OCR measurements

XF96 glycolysis stress test and mito stress test were performed using Seahorse XFe96 Extracellular Flux Analyzer (Agilent) to measure the extracellular acidification rate (ECAR) and oxygen consumption rate (OCR). One day prior to the assay, A549 cells were seeded 7000 cells/well in a 96-well Seahorse plate. A549 cells were treated with 100 nmol/L anti-B7-H3 antibody 8H9 or control antibody for 24 h. Before measurements, the growth medium was replaced with XF assay medium and the cells were incubated in a CO_2_-free incubator for 1 h. After the tests, cells were lysed with lysis buffer (0.1% triton, 10 mM Tris–HCl) and Bradford reagent was utilized to determine the total amount of protein in each well. Final concentrations of reagents utilized in the glycolysis stress were as follows: 10 mmol/L glucose, 2 μmol/L of oligomycin, and 50 mmol/L of 2-deoxy-D-glucose (2-DG) in each group. Final concentrations of reagents utilized in the mito stress test were as follows: 2 μmol/L of oligomycin, 2 μmol/L of FCCP, and 0.5 μmol/L of antimycin A/rotenone in each group.

### Intracellular ROS determination

Intracellular reactive oxygen species (ROS) was measured using 2,7-dichlorofluorescin diacetate as a probe. A549 cells were harvested and incubated with BiKE and control antibodies for 24 h. Then, cells were placed in growth medium, loaded with the probe for 15 min and then washed once with PBS. Fluorescence intensity was measured using the Accuri^™^ C6 flow cytometry.

### Statistical analysis

All statistical analyses were performed using GraphPad Prism software. Data are represented as mean $\pm$ standard deviation or individual values. Significant differences were calculated using the two-way ANOVA, Student’s ﻿t tests, nonparametric Mann–Whitney test, or log-rank test. P values are represented as: *p < 0.05, **p < 0.01, ***p < 0.001 and ****p < 0.0001.

## Results

### Broad expression of B7-H3 on different tumor cells and tissues

We first examined the expression of B7-H3 in a panel of tumor cell lines, including lung (A549, HCC827 and NCI-H23), breast (MDA-MA-231, BT-474 and HCC1954), colon (DLD-1 and HCT 116), ovarian (OVCAR-3 and SK-OV-3), acute myeloid leukemia (AML) (OCI-AML-3 and MOLM-13), pancreatic cancer (PANC-1), glioblastoma (SF188 and U251) and Burkitt's lymphoma (Daudi) cell lines, using flow cytometry and western blot assays. As shown in Additional file 1: Fig. S1A, immunofluorescent staining showed that B7-H3 was strongly expressed in tumor cell lines (A549, HCC827, HCT-116, DLD1, MDA-MB-231, OCI-AML-3, BT-474, OVCAR-3, SK-OV-3, PANC-1, HCC1954, SF188 and U251), while NCI-H23 and MOLM-13 cells were weakly stained. In contrast, B7-H3 expression was not detectable in Daudi cells that were used as the negative control, which is consistent with the previous study [41]. We observed that PD-L1 was highly expressed on the surface of B7-H3 + tumor cell lines, including NSCLC (A549), breast cancer (HCC1954), pancreatic cancer (PANC-1), glioblastoma (SF188 and U251) (Additional file 1: Fig. S2). Likewise, Western blot analysis results corroborated that majority of tumor cell lines were found to have a high level of B7-H3 expression, while NCI-H23 and MOLM-13 cells showed the relatively low level. (Additional file 1: Fig. S1B). B7-H3 was still undetectable in Daudi cells. The total amount of B7-H3 expressed in tumor cells was consistent with the results obtained in the cell surface analysis.

**Fig. 1:**
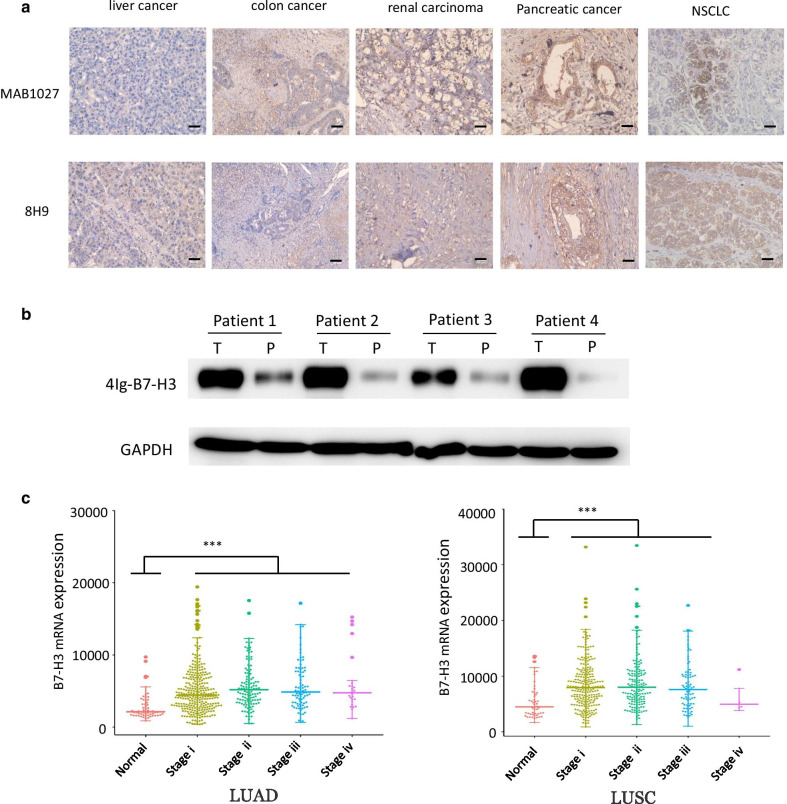
Expression of B7-H3 in human tissues. **a** ﻿IHC of FFPE tissues was used to evaluate B7-H3 expression in various human tumors. ﻿The sale bar represents 20 μm. **b** Western blot analysis of B7-H3 in tumor (T) and paracancerous (P) tissues from four NSCLC patients. The tissue lysates were probed with the goat anti-B7-H3 antibody. The levels of GAPDH were utilized as loading control. **c** Differential expression of B7-H3 in normal lung tissues, lung adenocarcinoma (LUAD) and squamous cell carcinoma (LUSC) in different stages was analyzed using RNA-seq datasets from TCGA. Significant differences were calculated using Student’ *t* test ﻿(*p < 0.05, **p < 0.01, ***p < 0.001)

To assess the expression levels of B7-H3 in human primary tissues, we next performed IHC staining on the different types of Formalin-fixed paraffin-embedded (FFPE) human tumor (lung cancer, colon cancer, ﻿hepatocellular carcinoma, pancreatic cancer, kidney cancer, melanoma) and normal tissues (lung, colon, liver, pancreas, kidney) with the anti-B7-H3 antibody 8H9 and a goat antihuman B7-H3 antibody from R&D Systems. IHC staining results showed that B7-H3 was widely expressed in all types of solid tumors with a high percentage (29/39, 74%) but was not detectable in most of the normal tissues (31/34, 91%). Two normal pancreatic and one kidney tissues showed the weak expression of B7-H3. ﻿ Of 24 lung cancer samples, 76% were positive for B7-H3, with 38% demonstrating high-intensity staining of 2 + and 3 + . ﻿No positive staining was noted in normal lung tissues. The details of the IHC results are provided in Table 1. Representative specimens are shown in Fig. 1a. ﻿Although 8H9 did not perform adequately when used as a primary antibody to stain FFPE tumor sections, the staining of 8H9 was specific for tumor tissues but not normal tissues. After being stained with an antihuman PD-L1 antibody, approximately 30% of B7-H3 + lung tumor samples showed positive (Additional file 1: Fig. S3). One pancreatic sample and one melanoma tumor sample showed positive for PD-L1. Moreover, the fresh tumor and paracancerous tissues were collected from four NSLCL patients and analyzed for B7-H3 expression using Western blot. It is noteworthy that in both tumor and surrounding tissues, B7-H3 showed high level of expression (Fig. 1b). We further evaluated the mRNA expression of B7-H3 in the patients with LUAD or LUSC with different stages using data from TCGA (Fig. 1c). We found that the mRNA levels of B7-H3 in LUAD (*p* < 0.001) and LUSC (*p* < 0.001) were significantly higher than those in the normal lung tissues. ﻿High B7-H3 expression was significantly associated with poor 5-year overall survival (5yr-OS) of patients with LUAD (*p* = 0.0037; Additional file 1: Fig. S4). Its high expression level had no significant effect on OS of patients with LUSC. Taken together, these experiments demonstrated that B7-H3 expression patterns were highly conserved in lung cancer.

**Table 1:** IHC staining of human normal and tumor tissues

Tissue	Number	Negative (%)	Positive (%)		
			+	+ +	+ + +
*Tumor*					
NSCLC	21	24	38	19	19
Colorectal	6	33	33	17	17
Liver	4	75	25	0	0
Melanoma	4	0	0	25	75
Pancreas	2	0	0	50	50
Renal	2	0	100	0	0
*Normal*					
Lung	10	100	0	0	0
Colon	8	100	0	0	0
Liver	4	100	0	0	0
Pancreas	10	80	20	0	0
Kidney	2	50	50	0	0

### B7-H3 CAR was effective to slow down NSCLC growth

**Fig. 2:**
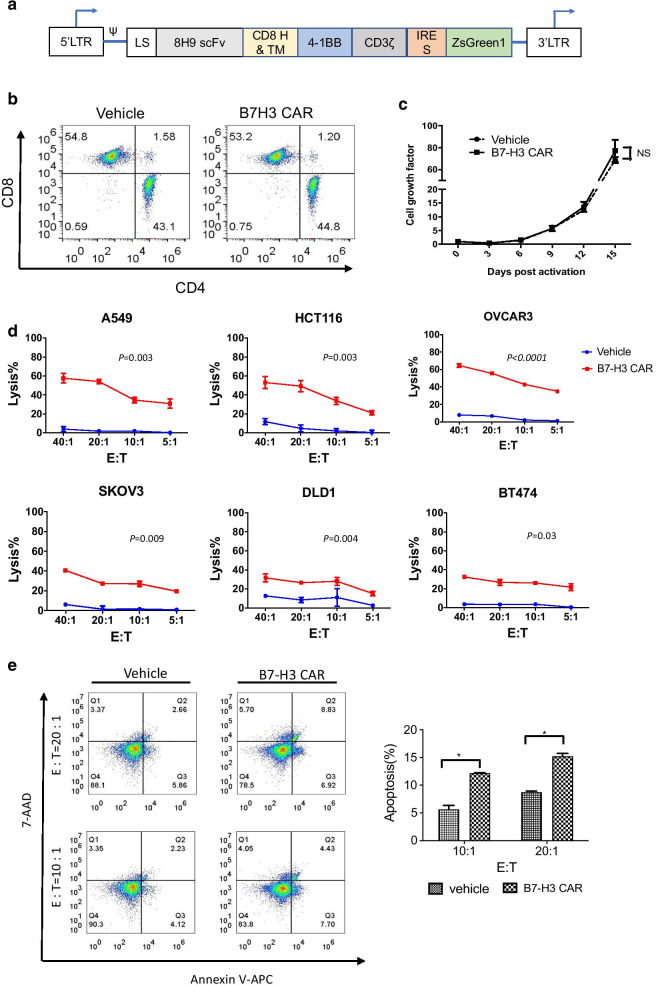
Generation of B7-H3 CAR- T cells and their in vitro antitumor activity. **a** Schematic diagram of the CAR construct. CAR gene was cloned into a lentiviral vector, which contained an internal ribosome entry site (IRES)-green fluorescence protein (ZsGreen) sequence. The resulting CAR was composed of the leader sequence (LS), a scFv, a hinge (H) region, a CD8α transmembrane domain (TM), along with the 4-1BB domain and the CD3ζ chain. **b** Subsets of B7-H3 CAR-T cells were derived from a single healthy donor. T cells were stained with anti-CD3, anti-CD4, or anti-CD8 antibodies and then analyzed using flow cytometry. **c** Proliferation of B7-H3 CAR-T cells and vehicle T cells activated by anti-CD3/CD28 beads. **d** Cytotoxicity of B7-H3 CAR-T cells (red line) and vehicle T cells (blue line) cells against various tumor cell lines. **e** Apoptosis in target A549 cells induced by B7-H3 CAR-T cells and vehicle T cells as control at different E:T ratios. Representative graphs are shown. The *p* values of the difference between the CAR group and the control group were analyzed using ANOVA

The B7-H3 CAR was generated by linking the scFv of anti-B7-H3 antibody 8H9 to the CD8 transmembrane domain, the intracellular 4-1BB co-stimulation with the CD3ζ signaling domain (Fig. 2a). ﻿The expression of the B7-H3 CAR on T cells was determined by detection of the co-expressed ZsGreen. Transduction efficiencies for the B7-H3 CAR and vehicle ranged from 60 to 75% in 293 T cells (Additional file 1: Fig. S5A) and human T cells (Additional file 1: Fig. S5B), respectively. After lentiviral transduction, the transduction efficacy of T cells ranged from 60–70% by detection of the report gene. The expanded cells showed high viability (above 95%). Compared with vehicle T cells, B7-H3 CAR-T cells showed no significant differences in neither CD4/CD8 ratios (Fig. 2b) nor proliferation (Fig. 2c). The results of quantitative PCR (qPCR) showed that the B7-H3 CAR was specifically expressed in human T cells (Additional file 1: Fig. S5C).

The in vitro cytotoxicity of B7-H3 CAR T cells was evaluated against six B7-H3-positive tumor cell lines (A549, HCT 116, OVCAR-3, SK-OV-3, DLD-1 and BT-474). The tumor cells were cocultured with B7-H3 CAR-T cells or vehicle T cells at a different effector/target (E: T) cell ratios. As shown in Fig. 2d, B7-H3 CAR T cells conferred antitumor lytic activity. All of B7-H3-positive tumor cell lines efficiently responded to the B7-H3 CAR. There was no obvious cytotoxicity of vehicle T cells at all E:T ratios. We have further analyzed apoptosis in A549 and HCT 116 cells induced by B7-H3 CAR-T cells. The percentage of tumor cells that underwent apoptosis was significantly higher in the presence of B7-H3 CAR-T cells than vehicle T cells (Fig. 2e).

**Fig. 3:**
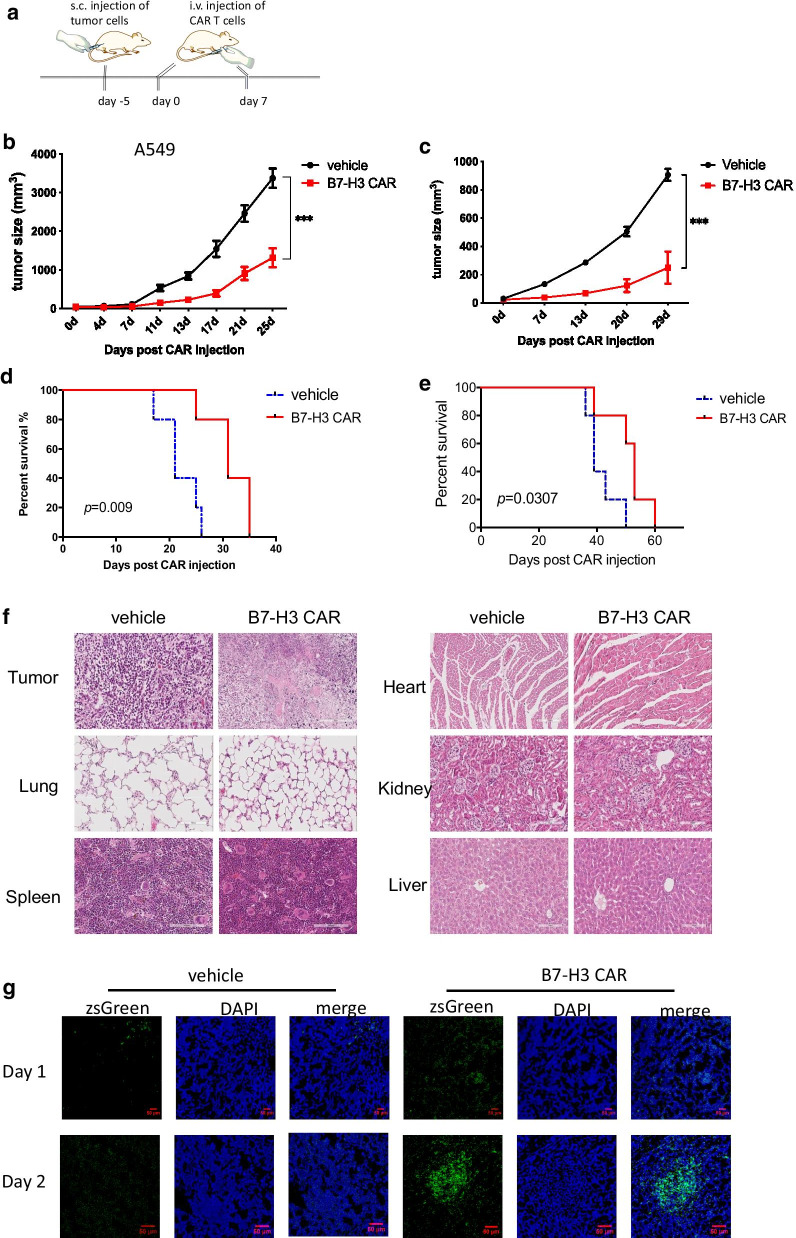
Therapeutic effects of B7-H3 CAR-T cells in tumor xenografts. **a** Treatment scheme of B7-H3 CAR-T cells in A549 or HCT 116 xenograft models. **b**–**e** ﻿Tumor growth in A549-injected mice **b** or HCT 116-injected mice **c** was measured using digital caliper, and tumor area was calculated (n = 5). ﻿Mean values per treatment group are shown. ﻿The *p* values of treatment were analyzed using ANOVA. Survival curves of A549-injected mice **d** or HCT 116-injected mice **e** are shown. ﻿The *p* values of survival curves were analyzed using the log-rank (Mantel–Cox) test. ﻿Data are presented as the mean ± standard deviation. *p < 0.05, **p < 0.01, ***p < 0.001. **f** H&E staining of major organs in mice treated with CAR-T cells. **g** ﻿ Tumor infiltration of B7-H3 CAR-T cells in A549-injected mice. ﻿On days 1 and 2, the tumors were harvested, sliced, fixed and imaged in the fluorescence microscope. ﻿Representative images are shown. ZsGreen is used as the reporter of CAR-T cells. ﻿Representative images are shown

The in vivo antitumor efficacy of B7-H3 CAR T cells was evaluated using xenograft mouse models of NSCLC (A549) and colorectal cancer (HCT 116). Subcutaneous xenotransplanted tumor models were established in NOD/SCID mice. The mice were administered with ﻿ two infusions of B7-H3 CAR or vehicle T cells intravenously (i.v.) via tail vein on day 0 and day 7, respectively (Fig. 3a). In xenograft models of A549 (Fig. 3b, d) and HCT 116 (Fig. 3c, e), B7-H3 CAR-T cells successfully delayed tumor growth and prolonged survival in animals compared with vehicle T cells. No significant weight loss was observed in mice (Additional file 1: Fig. S6A–B). No treatment-related adverse effects were observed in all groups treated with B7-H3 CAR-T cells. H&E staining showed that ﻿no evident lesions were formed in major organs collected from mice that were treated with B7-H3 CAR-T compared with the control group (Fig. 3f). The results suggested that B7-H3 CAR-T cells had no obvious systemic acute toxicity in major organs that were harvested from A549-injected mice. Overall, the B7-H3 CAR was effective to delay tumor growth in tumor-bearing mice without causing detectable organ toxicity.

### B7-H3 redirection enhanced T cell functional properties and tumor infiltration

**Fig. 4:**
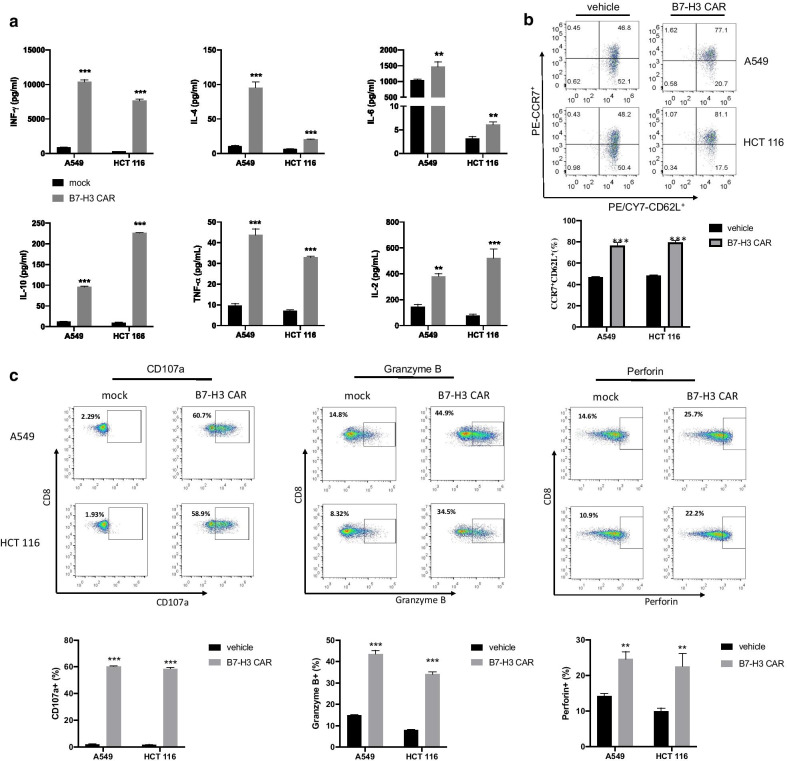
Functional analysis of B7-H3 CAR-T cells. **a** Cytokine production in B7-H3 CAR and vehicle T cells. Effector T cells were incubated with target A549 or HCT 116 cells at a ratio of 10:1 for 24 h. ﻿The concentrations of cytokines were measured using flow cytometry. **b** Memory cell phenotypes of B7-H3 CAR-T cells. Effector T cells were incubated with target A549 or HCT 116 cells at a ratio of 10:1 for 24 h. Expression of CD62L and CCR7 on T cells was identified with staining with anti-CD62L and anti-CCR7 antibodies, respectively, and measured using flow cytometry. **c** Degranulation markers in B7-H3 CAR T cells. ﻿Effector T cells were incubated with target A549 or HCT 116 cells at a ratio of 10:1 for 6 h (CD107a) or 24 h (granzyme B and perforin). Expressions of CD107a, intracellular granzyme B and perforin staining on T cells were stained with anti CD107a, granzyme B and perforin antibodies and measured by flow cytometry. Data from 2–3 independent experiments are presented as the mean ± standard deviation. *p < 0.05, **p < 0.01, ***p < 0.001. The *p* values were analyzed using a Student’ *t* test

﻿We assessed key cytokine secretion of B7-H3 CAR-T cells in vitro. After 24-h co-incubation of tumor cells and effector T cells at an E:T ratio of 10:1, B7-H3 CAR-T cells produced significantly higher amounts of IFN-γ, TNF-α, IL-2, IL-6, IL-4 and IL-10 in the culture supernatants compared with vehicle T cells (Fig. 4a). To further determine CAR-T cell phenotype, we examined the functional status of CD8^+^ T cells that were in contact with target cells. B7-H3 CAR-T cells exhibited the increased proportion of CCR7^+^CD62L^+^ cells compared with vehicle control T cells, suggesting that B7-H3 CAR-T cells differentiated into the naïve and central memory T cells ﻿which are considered as favorable phenotypic characteristics to ensure persistence and antitumor effects (Fig. 4b). Three degranulation markers, namely CD107a, granzyme B and perforin, were detected on cell surface of B7-H3 CAR-T cells. As shown in Fig. 4c, there was a significantly increased level of CD107a expression in B7-H3 CAR-T cells compared with that in control T cells. We further demonstrated that intracellular granzyme B and perforin levels in B7-H3 CAR T cells were higher than those in vehicle T cells by flow cytometry (Fig. 4c). The results of qPCR further verified that the total mRNA level of granzyme B was significantly increased in B7-H3 CAR T cells (Additional file 1: Fig. S7). Therefore, B7-H3 CAR-T cells possibly exert cytotoxic antitumor effects through a degranulation-mediated mechanism.

To further analyze the in vivo mechanism of B7-H3 CAR action, we performed fluorescent imaging analysis on tumors and organs excised from the mice that were undergoing treatment. Mice were killed at 1–2-day post-injection of B7-H3 CAR-T and vehicle T cells. ﻿Tumors and major organs were excised, fixed, sliced and subjected to fluorescence microscopy observation. In contrast to the control group, the B7-H3 CAR-treated tumor samples exhibited T cell infiltration throughout the tissue at 2-day after treatment (Fig. 3g). Other major organs, such as the heart, spleen, lung, liver and kidney, did not display obvious cross-reactivity with CAR-T cell (Additional file 1: Fig. S8). ﻿These data ﻿corroborate that B7-H3 redirection promotes T cell to infiltrate into tumors in a specific manner.

### B7-H3/CD16 BiKE induced antitumor efficacy mediated by NK cells

**Fig. 5:**
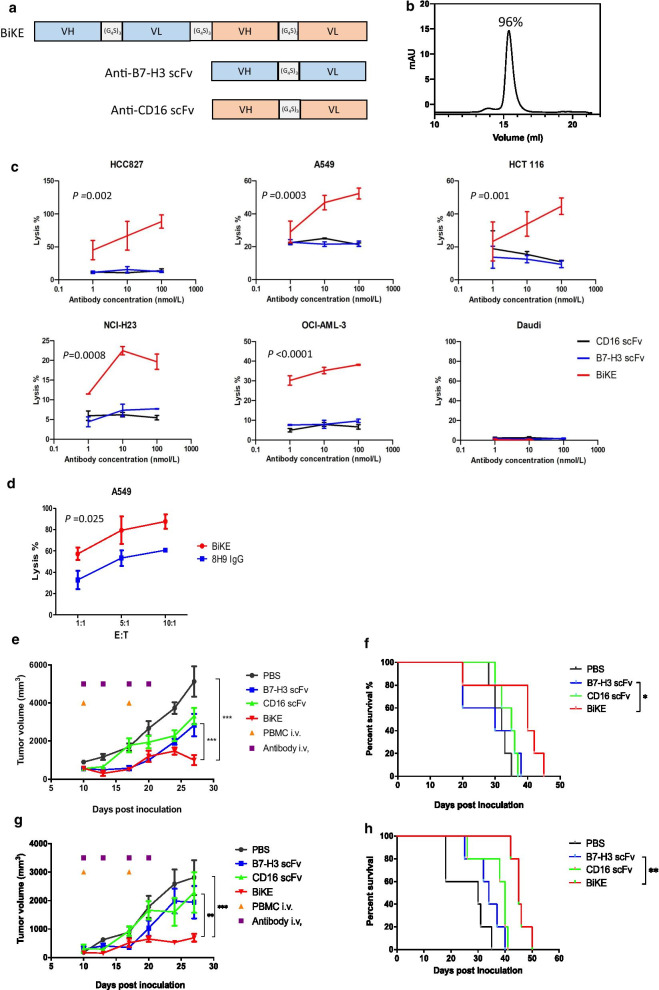
B7-H3/CD16 BiKE induced antitumor effect mediated by NK cells *in vitro* and in vivo. **a** Construct diagrams of B7-H3/CD16 BiKE, anti-B7-H3 scFv, and anti-CD16 scFv. **b** Purity of B7-H3/CD16 BiKE determined by size-exclusion chromatography. **c** Cytotoxicity of B7-H3/CD16 BiKE (red line), anti-B7-H3 scFv (blue line) and anti-CD16 scFv (black line) against different tumor cell lines by PBMC as effectors. **d** Comparison of ADCC induced by B7-H3/CD16 BiKE and anti-B7-H3 IgG 8H9. PBMCs were used as effectors and A549 cells were used as targets. Representative graphs are shown. For the experiments **c**–**d**, the *p* values of the difference between BiKE and control groups were analyzed using ANOVA. **e**–**h** Therapeutic effects of B7-H3/CD16 BiKE in A549 and NCI-H23 xenografted mice. Antibodies were administered (100 μg per dose) intravenously twice a week for a total of four doses. Human PBMCs (10 million per iv injection) were delivered intravenously twice. Tumor growth in A549-injected mice **e** or HCT 116-injected mice **g** was measured using digital caliper, and tumor area was calculated (n = 5). Mean values per treatment group are shown. ﻿ Survival curves of A549-injected mice **f** or HCT 116-injected mice (**h**) are shown. The *p* values of treatment were analyzed using ANOVA. The *p* values of survival curves were analyzed using the log-rank (Mantel–Cox) test. ﻿

The B7-H3/CD16 BiKE, which simultaneously targets B7-H3 and a NK cell activator CD16, was constructed by linking the anti-B7-H3 8H9 scFv and the anti-CD16 scFv with a nonimmunogenic (glycine_4_—serine)_3_ linker (Fig. 5a). A C-terminal 6 × His-tag was added for purification of the protein products. The BiKE was expressed in Freestyle 293-F cells and purified using nickel-NTA resins. Meanwhile, the anti-B7-H3 scFv and anti-CD16 scFv antibodies were produced and purified. The molecular weight and purity of the BiKE protein and scFvs were shown using SDS-PAGE (Additional file 1: Fig. S9B) and SEC (Fig. 5b). The purified B7-H3/CD16 BiKE exhibited the binding ability to both soluble 4Ig-B7-H3 and CD16a proteins in ELISA assay (Additional file 1: Fig. S9C-D). BiKE showed comparable binding affinity to 4Ig-B7-H3 protein compared with the 8H9 scFv as previously reported (scFv, K_D_ = 6.71 nM vs. BiKE, K_D_ = 2.94 nM, Additional file 1: Table S1). Flow cytometry analysis further demonstrated that BiKE recognized CD16 expressed on the cell surface of NK-92MI cells (Additional file 1: Fig. S9E). In a transwell migration assay, CD16a-expressing NK-92 cells and B7-H3-positive A549 cells were seeded at upper and lower champers, respectively. NK cell migration was significantly enhanced after the treatment with B7-H3/CD16 BiKE (Additional file 1: Fig. S10).

To determine the antitumor potency of the BiKE, ADCC assay was performed by co-incubating the effector PBMCs with the solid tumor cell lines (A549, HCC827, NCI-H23, HCT 116), AML cell lines (OCI-AML-3) and negative control cell lines (Daudi) at the presence of antibodies with different concentrations. B7-H3/CD16 BiKE-mediated cytotoxicity was specific as the BiKE significantly ﻿potentiated cytotoxic activity against B7-H3-positive solid tumor cells, whereas no cell death was observed in a B7-H3-negative cell line Daudi (Fig. 5c). Although NCI-H23 weakly expressed B7-H3, it still displayed the cytotoxicity response to BiKE. Meanwhile, specific lysis was also observed in AML, OCI-AML-3. As controls, the anti-B7-H3 and anti-CD16 scFv had no significant effects on the increase of cell lysis function. Although anti-B7-H3 8H9 IgG showed the ability to induced ADCC activity [26], we found that BiKE exerted greater ADCC ability than anti-B7-H3 8H9 IgG (Fig. 5d), ﻿suggesting that the BiKE may be more efficient at recruiting effector NK cells for targeting cell lysis. Therefore, the addition of B7-H3/CD16 BiKE to the cell culture resulted in significant NK cell cytolytic activity.

We next tested the in vivo efficacy of the BiKE in A549 and NCI-H23 xenograft NOC/SCID mice model with human PBMC infusion. Intravenous injections of the BiKE and scFvs were done twice weekly for 2 weeks. In the first experiment with A549, the BiKE was able to reduce tumor volume area under the curve (AUC) by 80% compared with that in the PBMC treated group (*p* < 0.001) (Fig. 5e). In the second experiment with NCI-H23, ﻿the significant tumor growth repressions were observed in the BiKE group compared with PBMC (*p* < 0.001) and anti-B7-H3 scFv (*p* < 0.05) (Fig. 5g), respectively. Tumor volume AUC was reduced under the curve by 75%. Significantly prolonged survival was observed in the BiKE-treated groups compared with that in the group of anti-B7-H3 scFv in two experiments (Fig. 5f, h). No significant weight loss was observed in mice treated with antibodies throughout the course of this study (Additional file 1: Fig. S6C). H&E staining showed that ﻿no evident lesions were formed in major organs collected from mice that were treated with the BiKE (Additional file 1: Fig. S11A). The serum levels of hepatic enzymes, alanine transaminase (ALT) and aspartate transaminase (AST), were measured. The activities of these enzymes are usually elevated during damage to liver cells and tissues. No significant difference was observed between the treatment and PBS-treatment groups (Additional file 1: Fig. S11B). The results suggested that the BiKE had no obvious systemic acute toxicity in major organs.

**Fig. 6:**
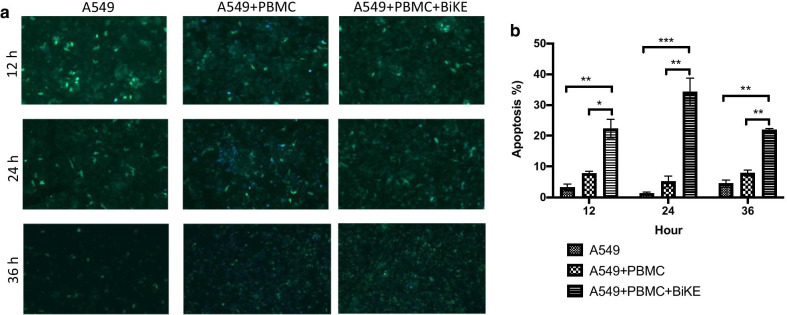
Dynamic caspase-3 activation in apoptotic cells in response to B7-H3/CD16 BiKE. **a** FRET imaging of A549-C3 cells live cells appeared in green color and apoptotic cells in blue color. A549-C3 cells were treated with B7-H3/CD16 BiKE (5 μg/mL) with PBMCs at E:T = 10 for 12,24, and 36 h. **b** Statistical analysis of apoptotic rate at different time points. Representative images are shown. Sample size: 1 × 10^5^ target cells. The *p* values were analyzed using a Student’ *t* test

NK cell as the innate effector cell induces apoptotic cell death. To quantify BiKE-mediated tumor apoptosis induced by NK cells, we employed one a FRET-based quantitative live-cell imaging system. A549 cells expressing a caspase-3 biosensor FRET reporter (A549-C3), which indicates live cells with green fluorescence light and apoptotic cells with blue fluorescence light, were loaded with BiKE and human NK cells in the range of 12–36 h. The representative images of fluorescence of and apoptosis percentages in tumor cells are shown in Fig. 6a, b. At different time points, the BiKE induced cell apoptosis in target tumor cells mediated by NK cells, ranging from 23 to 35% of the total target cell population with statistically significant differences. Therefore, the results suggested that the recruitment of NK cells with BiKE enhanced apoptosis in target tumor cells.

### B7-H3 blockade altered glucose metabolism in cancer cells

**Fig. 7:**
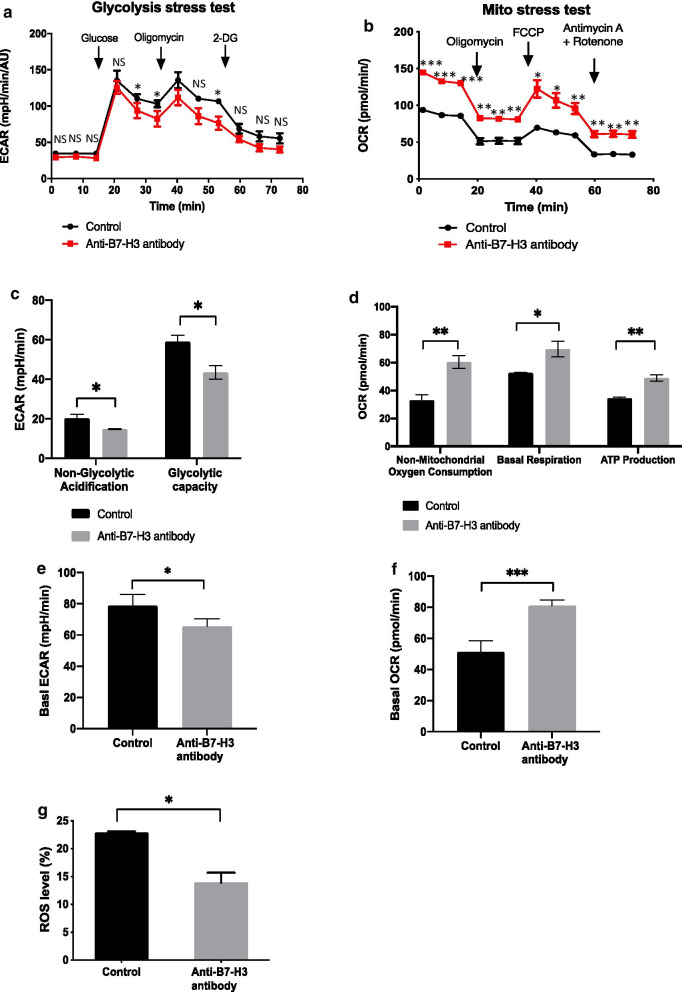
Anti-B7-H3 blockade influences on glucose metabolism and ﻿intracellular ROS in A549 tumor cells. **a**–**f** Extracellular acidification rate and oxygen consumption rate measurements using the seahorse extracellular flux analyzer. After A549 cells were treated with either anti-B7-H3 or control antibodies for 24 h, ECAR and OCAR were examined using the Mito Stress Cell and Glycolysis Stress assays. ECAR (**a**, **e)** and OCR (**b**, **f**) metabolic profiles are shown. **c** Nonglycolytic acidification and glycolytic capacity were derived from Glycolysis Stress assay results. **d** Nonmitochondrial respiration, ATP production and basal respiration were derived from Mito Stress Cell results. **g** Intracellular ROS measurement in tumor cells. A549 cells were labeled with 2,7-dichlorofluorescin diacetate as a probe and treated with either anti-B7-H3 or control antibodies. Intracellular ROS signals were measured using flow cytometry. Data from two independent experiments are presented as the mean ± standard deviation. ﻿(*p < 0.05, **p < 0.01, ***p < 0.001). The *p* values were analyzed using a Student’ t test

﻿ Warburg effect is one feature of metabolic dysregulation during malignant transformation. To investigate the effect of anti-B7-H3 blockade in the regulation of ﻿glucose metabolism in cancer cells, we measured dynamic ECAR and OCR as indicatives of ﻿glycolysis and ﻿oxidative respiration in A549 cells. ﻿The parameters of mitochondrial respiration and glycolytic capacity of cancer cells were determined using the Mito Cell Stress and the Glycolysis Stress assays, respectively. We found that anti-B7-H3 blockade decreased basal ECAR (Fig. 7a, e) and increased basal OCR (Fig. 7b, f). ECAR was low while OCR was high, reflecting indicatives of anti-Warburg effect. ECAR was increased, reflecting the inhibition for nonglycolytic acidification and glycolytic reserve capacity (Fig. 7c), and OCR was decreased, reflecting the inhibition a greater requirement of nonmitochondrial oxygen consumption, basal respiration and ATP production oxidative phosphorylation (Fig. 7d). The cells produced ATP primarily by oxidative phosphorylation. The results suggest that anti-B7-H3 blockade switches cell metabolism from glycolysis to oxidative phosphorylation. Because ROS plays essential roles in modulating glucose metabolism, we further determined whether ROS levels could be altered by anti-B7-H3 blockade in A549 cells. The cellular ROS levels in A549 cells under anti-B7-H3 antibody treatment were measured by staining with fluorescent probes. We found that the treatment with anti-B7-H3 antibody resulted in lower levels of ROS (Fig. 7G and Additional file 1: Fig. S12). Thus, ﻿anti-B7-H3 blockade probably regulates glucose metabolism through cellular ROS signals.

## Discussion

﻿Despite great progress in the development of immune checkpoint inhibitors, particularly the encouraging therapy of PD-L1/PD-1 blockade, a portion of NSCLC patients remain generally resistant to these therapies [42]. There is an ongoing research aimed to discover novel immune checkpoint biomarkers and develop novel immunotherapy approaches. Overexpression of B7-H3 in tumors and its low levels in normal tissues have been described [14, 43]. Recent studies have demonstrated that B7-H3 overexpression was associated with poor prognosis in NSCLC patients [16, 17]. Consistently, this study showed that ﻿B7-H3 overexpression was significantly associated with 5yr-OS of NSCLC patients. We demonstrated that B7-H3 was tightly expressed in human NSCLC. The expression of B7-H3 in tumor and normal tissues was analyzed based on the data from the TCGA and IHC. According to the TCGA data, the mRNA expression levels of B7-H3 were highly associated with the malignancy grade of NSCLC. The IHC results further showed that 76% of NSCLC tissues were positive for B7-H3, which was consistent with the previous studies [18]. B7-H3 expression may be correlated with tumor metastasis, because moderate expression of B7-H3 was also observed in paracancerous tissues, although the intensity was weaker than that in tumor tissues. Thus, this finding has further confirmed utility of B7-H3 as an oncoimmunology target.

Although the regulatory mechanisms of B7-H3 have not been completely elucidated, ﻿ B7-H3 function closely correlates with that of a cytotoxic lymphocyte. As described in previous studies, anti-B7-H3 antibody blockade augmented antitumor immunity of CD8^+^ T cells and NK cells [12, 18]. The anti-B7-H3 mAb 8H9 (omburtamab) has showed clinical potential in promoting Fc-dependent NK cell through ADCC [26]. NK cells are the key component in ADCC and play a role in immunosurveillance and the prevention of tumor metastasis [44, 45]. Unlike T cell, NK cells are devoid of receptors for tumor-associated antigens. Solid tumors are relatively resistant to NK cytotoxicity due to lack of NK activation ligands [46]. Tumor metastasis is often associated with a low NK cell activity [47, 48]. NK dysfunctions promote metastasis in several human malignancies [49, 50]. In this study, to overcome resistance, we examined the ability of the B7-H3/CD16 BiKE to redirect NK cells to destroy tumor cells. In fact, BiKE triggers activation of resting NK cells through CD16a signaling and thereby induces the secretion of cytokines and degranulation against B7-H3-positive tumor cells. Importantly, due to the direct binding of antibody to CD16a, NK cell cytotoxicity induced by the BiKE ﻿was superior to the Fc-mediated ADCC of anti-B7-H3 8H9 IgG. In two xenografts involving A549 (high level of B7-H3) and NCI-H23 (low level of B7-H3), our results showed that BiKE was effective to suppress NSCLC growth in both models as was demonstrated with reduced tumor volume (75–80%), The in vivo activity of BiKE was not limited by B7-H3 antigen density of NCI-H23 at the low level. We assume that a threshold of B7-H3 density is required to induce NK activity. The NK cell effector mechanisms involve caspase-dependent apoptosis [51]. Dynamics of tumor cell death induced by NK cells was observed using FRET-based quantitative live cell imaging system in which activation of caspase-3 was used as indication of apoptosis. As NK stimulation by BiKE leads to significant increases in apoptotic target cell death, this further confirmed ﻿the ability of BiKE to actively propagate signals for NK cell activation.

In order to exploit the potential of 8H9 as CAR, we described the effects of the B7-H3 CAR-based 8H9 in cytotoxicity, cytokine production and inhibition of tumor growth. Our results demonstrated that key cytokine levels and T cell degranulation were enhanced. B7-H3 blockade promoted T cells differentiation into the naïve and central memory T cells, ﻿reflecting the increase of T cell persistence. Indeed, the B7-H3 CAR of 8H9 showed significant antitumor activity in xenografts of NSCLC and colon cancer cells. ﻿The on-target off-tumor toxicity is a major safety concern in CAR-T therapy [52]. Efforts have been made to limit the toxicity, including infusing low dose of CAR-T cells [53] and optimizing antibody affinity for the target [54]. Our results showed that the B7-H3 CARs are expected to have minimal off-target toxicity. Because B7-H3 expression is limited in normal tissues, B7-H3 CAR T cells mainly extravasated into solid tumors but not into normal tissues. ﻿Consistent with this observation, we did not find evidence of damage in normal tissues. It suggests that tumor regional infiltration of B7-H3 CAR-T cells may limit systemic toxicity by reducing the cross-reactivity of CAR-T cells in other organs.

Combination strategies of immune checkpoint inhibitors provide significant benefit to some cancer patients [55, 56]. In NSCLC patients, ﻿B7-H3 was associated with adaptive resistance to anti-PD-1 therapy [18]. An anti–B7-H3 mAb, MGA271, has been assessed in a Phase I trial (NCT02475213) in combination with anti-PD-1 antibodies (pembrolizumab) in patients with melanoma. Combining blockades of B7-H3 and PD-1/PD-L1 resulted in the strength of therapeutic effects mediated by CD8 + T cells in the mouse ovarian cancer and pancreatic cancer models [12, 18, 57]. In this study, IHC staining showed that approximately 30% of patients with NSCLC had co-expression of B7-H3 and PD-L1. On the other hand, a recent study demonstrated that ﻿﻿PD-L1 expression by T cells weakened antitumor immunity through suppressing effector T cells and macrophages in tumor microenvironment [58]. In our studies, PD-L1 expression was found in B7-H3 CAR-T cells irrespective of PD-L1 expression in tumor cells (Additional file 1: Fig. S13). We speculate that T-cell-expressed PD-L1 is involved in modulation of B7-H3 CAR-T cells. In the preliminary experiments, one anti-PD-L1 antibody improved cytotoxicity of B7-H3 CAR T cells at the high E:T ratio of 40:1 (Additional file 1: Fig. S14), suggesting anti-PD-L1 blockade should be able to prevent ﻿the immune-suppressive signaling of PD-L1 expressed by either tumor cells or T cells. In other experiments, we observed the increased productions of IL-2 and TNF-α by CAR T cells that were treated with anti-PD-L1 antibody (unpublished data). However, the role of PD-L1 has not yet been defined for B7-H3 CAR T cells. Future studies are needed to determine whether there is a correlation between T cell-associated PD-L1 and antitumor effects of B7-H3 CAR T cells in vivo. Unlike in T cells, PD-1 is not highly expressed in NK cells [59]. In contrast, BiKE effectively controlled tumor growth by modulating NK cells probably due to the limited PD-1 levels. Therefore, it may be interesting to investigate further the therapeutic efficacies of dual blockade of B7-H3 and PD-L1 in NSCLC.

Elicitation of cellular mechanism behind the anti-B7-H3 blockade is important to reveal the immune-independent functions of B7-H3. The Warburg effect is a metabolic hallmark of tumor cells ﻿ [60]. Although recent studies suggested that B7-H3 modulated glucose metabolism in breast cancer and colorectal cancer [61], the effects of B7-H3 on aerobic glycolysis in NSCLC remain unknown. Unlike other anti-B7-H3 antibodies, 8H9 can recognize the FG loop of B7-H3 [26], which may be related to the regulatory functions of B7-H3. ﻿Here, we demonstrated that anti-B7-H3 blockade by 8H9 antibody switched cell metabolism from glycolysis to oxidative phosphorylation. The alteration of glucose metabolism probably was associated with cellular ROS-mediated pathway, consistent with the previous reports [61]. These results clearly showed that, in addition to immune-regulatory function, B7-H3 possessed immune-independent functions in NSCLC.

In summary, our study shows that B7-H3 is an attractive target in NSCLC since it is highly expressed in 76% of the tumor specimens. Recent advances and positive clinical results from bispecific antibodies and CAR T cells have shown hope and excitement. Two treatment modalities, namely the B7-H3 CAR and BiKE derived from the anti-B7-H3 mAb 8H9, provided an effectively control of the tumor growth in NSCLC through promoting immune cell response. Safety and immunotoxicity are considered for these immunomodulatory therapeutics. Although CAR T-cell therapy has demonstrated significant antitumor activity, it can be frequently associated with cytokine release syndrome (CRS). A significant advantage of BiKE is to present low risks of systemic toxicity [62]. In addition, another consideration is off-target toxicity that can cause dangerous side effects and is a major cause of clinical trial failure. Therefore, further research should explore the potential of anti-B7-H3 CAR and BiKE against NSCLC in the future preclinical and clinical studies.

## Supplementary Information

**Additional file 1.** Supplementary method, figure and table.

## Data Availability

All relevant data are within the paper and its Supplementary files.

## Abbreviations

ADCC: Antibody-dependent cell-mediated cytotoxicity
AUC: Area under the curve
NSCLC: Non-small cell lung cancer
CAR: Chimeric antigen receptor
BiKE: Bispecific killer cell engager
NK: Natural killer
PD-1: Programmed cell death protein 1
TCGA: The Cancer Genome Atlas
LUAD: Lung adenocarcinoma
LUSC: Lung squamous cell carcinoma
FFPE: Formalin-fixed paraffin-embedded
5yr-OS: 5-Year overall survival
scFv: Single chain variable fragment
VH: Heavy chain variable region
VL: Light chain variable region
MOI: Multiplicity of infection
MFI: Mean fluorescence intensity
FRET: Fluorescence resonance energy transfer
IHC: Immunohistochemistry
ECAR: Extracellular acidification rate
OCR: Oxygen consumption rate
2-DG: 2-deoxy-D-glucose
ROS: Reactive oxygen species
ALT: Alanine transaminase
AST: Aspartate transaminase
CRS: Cytokine release syndrome
