# Supplementary material for: Targeting B7-H3 via chimeric antigen receptor T cells and bispecific killer cell engagers augments antitumor response of cytotoxic lymphocytes
Source: J Hematol Oncol. 2021 Jan 29;14:21. doi: 10.1186/s13045-020-01024-8 (PMC7844995; doi:10.1186/s13045-020-01024-8)
Supplement: Supplementary file 1 — Additional file 1. Supplementary method, figure and table. [file 13045_2020_1024_MOESM1_ESM.pdf]

Supplementary Methods

Production anti-B7-H3 IgG 8H9

The heavy and light chain sequences of the anti-B7-H3 antibody 8H9 were cloned into the mammalian expression vector. For expression, 20 μL of plasmids (1 μg/mL) and 40 μL of PEI transfection reagents (1 μg/mL) were mixed in 2 mL endotoxin-free PBS and incubated for 10 minutes at room temperature (RT). Then the mixture was dorpwised to FreeStyle 293-F cells in 30mL FreeStyle 293 expression medium. After four days of post-transfection, the culture supernatant was harvested. Antibodies were purified by the Protein A Sepharose TM chromatography (GE Healthcare). The protein purity was examined by 12% SDS-PAGE and Western blot. The heavy chain of sequence is QVQLQQSGAELVKPGASVKLSCKTSGYTFTNYDINWVRQRPGQGLEWIGWIFPGDG STQYNEKFKGKATLTTDTSSSTAYMQLSRLTSEDSAVYFCARQTTGTWFAYWGQGT LVTVSA, and the light chain sequence is DIVMTQSPATLSVTPGDRVTLSCRASQSISDYLYWYQQKSHESPRLLIKYASQSISGIP SRFSGSGSGSDFTLSINSVEPEDVGVYYCQNGHSFPLTFGAGTKLELKR.

Size-exclusion chromatography (SEC)

The purified B7-H3/CD16 BiKE was loaded into the Superdex 200 increase 10/300 column (GE Healthcare) running on AKTA Pure system (GE Healthcare) to assess antibody purity.

Enzyme-linked immunosorbent (ESLIA) assay

Briefly, 100 ng B7-H3 protein (R&D system) or CD16a protein (Sino Biologicals) was coated on the 96-well ELISA plates (Nunc) at 4 ^{o}C overnight. The wells were blocked with PBS buffering containing 4% non-fat milk for 1h at RT. B7-H3 protein was bound with serial dilutions of anti-B7-H3 8H9 IgG, BiKE and anti-B7-H3 8H9 scFv at RT for 1h, followed by the detection by the HRP conjugated goat anti-Human IgG-Fc secondary antibody (Jackson ImmunoRes), anti-c-myc antibody (AbD Serotec), or the HRP conjugated anti-Flag tag antibody (Sigma) as the secondary antibody for 1h at RT. CD16a protein was bound with serial dilutions of BiKE and anti-CD16 scFv at RT for 1h, followed by the detection by the anti-c-myc antibody, or the HRP conjugated anti-Flag tag antibody (LifeTein) as the secondary antibody for 1h at RT. The anti-c-myc antibody was detected by HRP conjugated goat anti-mouse IgG antibody (Jackson ImmunoRes). The 3′,5,5′-Tetramethylbenzidine (TMB) (Beyotime) as the substrate was added and the reaction was read at 450 nm.

Bio-layer interferometry (BLI) assays

The binding affinities of B7-H3/CD16 BiKE and anti-B7-H3 scFv were performed by BLI on an Octet-K2 (Fortebio) using anti-mouse Fc (AMC) biosensors. 4IgB7-H3-mouse Fc protein with PBS containing 0.02% Tween 20 (PBST) was immobilized on the AMC biosensors from 1 to 4 nm. Then, 3-fold serial diluted antibodies in PBST were loaded for association and dissociation. Results were analyzed by ForteBio Data Analysis software.

Transwell migration assay

Approximately 1×10^5 NK-92-CD16a cells expressing eGFP were treated with 10 μg/mL BiKE or control scFv for 30 followed by seeding at the upper champers of the 24-well transwell plates (3 μm, Corning). 1×10^4 A549 cells were seeded into the lower chambers. After incubating plates for 16 h, cells were harvested from the lower side of the membrane. The migrated cells were counted using the BD Accuri™ C6 Flow Cytometer.

Western blot

Harvested tumor cells were lysed in radioimmunoprecipitation (RIPA) buffer supplemented with protease inhibitor cocktail (Promega). Protein concentrations were determined with a BCA protein assay. The proteins were separated in the SDS-PAGE gel, and transferred onto the PVDF membrane (Bio-Rad). After incubation with 5% non-fat milk for 1 h, the membrane was probed with the goat anti-human B7-H3 primary antibody (R&D Systems) overnight at 4 °C. The membrane was then incubated with the secondary HRP-conjugated rabbit anti-goat antibody (Jackson ImmunoRes) for 1 h at RT and visualized using Clarity Western ECL Substrate (Bio-Rad).

Quantitative real-time PCR (qPCR)

Total RNA samples were isolated from T cells using Trizol reagent (Invitrogen) according to the manufacturer's instructions. cDNA was generated from 2.5μg of RNA using iScript cDNA Synthesis Kit (Bio-Rad) according to the manufacturer's instructions. qPCR was performed with Bio-Rad CFX96 by using iTaq™ Universal SYBR Green (Bio-Rad). GAPDH was used as normalizers. The primers were listed as follows: granzyme B: forward 5'- GAGATCATCGGGGGACATGAG-3' and reverse 5'-AGCACGAAGTCGTCTCGTAT-3'; GAPDH: forward 5'-CACCGTCAAGGCTGAGA ACG-3' and reverse 5'-CATGGTGGTGAAGACGCCA-3'; CAR: forward 5'-GACCACCGA TACAAGCAGCA-3' and reverse 5'-GGCTCTG GGTCATCACGATA-3'. The average Ct values of the triplicate reactions were used for data analysis.

Haemotoxylin and eosin (H&E) staining

The tumor, liver, spleen, heart, lung, and kidney organs were collected from the sacrificed mice. After the fixation in 4% paraformaldehyde overnight at RT and three-time washes with PBS, the samples were dehydrated and embedded in paraffin. The paraffin-embedded tissue blocks were sliced into 5 μm sections. After dewaxed and washed, the sections were stained with hematoxylin and eosin. The slides were scanned with LEICA SCN400F Whole Slide Scanner. Data were analyzed with the software of Aperio ImageScope.

Immunofluorescent imaging

Tumor tissues were collected from mice that received CART cells treatment in day 1 and day 2, respectively. The tumor tissues were embedded with optimal cutting temperature (OCT) compound after fixed in 4% paraformaldehyde for 6 h at RT. After being frozen at -80°C overnight, tumor tissues were sliced into 10 μm sections using the LEICA CM5030 Cryostat and subjected to DAPI staining for observation. The slides were scanned with LEICA SCN400F Whole Slide Scanner. Data were analyzed with the software of Aperio ImageScope.

AST and ALT measurement

Serum levels of AST and ALT were measured using the commercial kits by Kiang Wu Hospital.

Supplementary Figures and Table

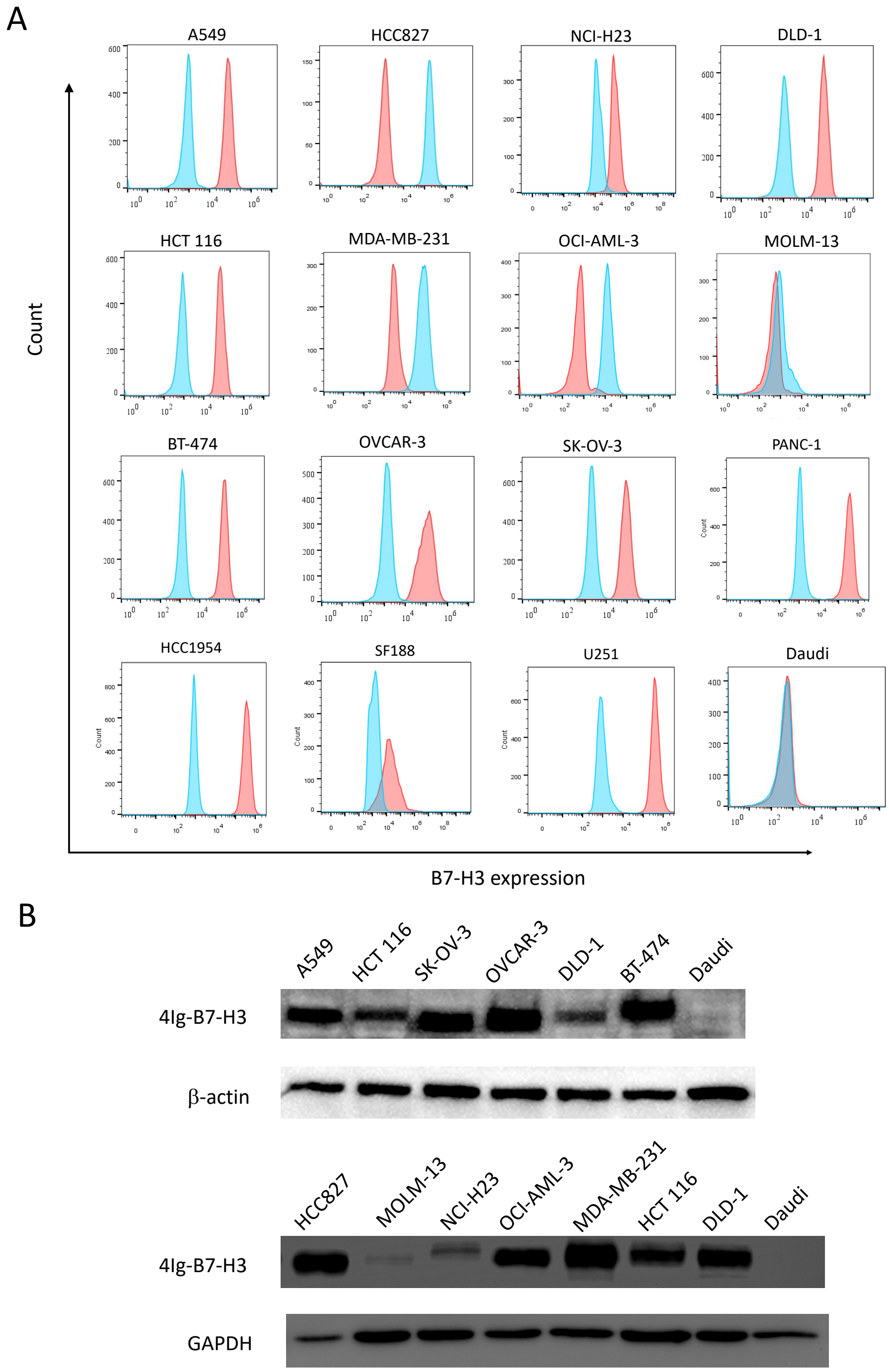

Figure S1. Expression of B7-H3 in human tumor cell lines. (A) Flow cytometric analysis of B7-H3 expression on the surface of tumor cells line. Immunostaining was performed using anti-B7-H3 IgG 8H9. Red color represents 8H9 IgG staining. Blue color represents control IgG staining. (B) Western blot analysis of B7-H3 protein in tumor cell lines. The cell lysates were probed by the goat anti-B7-H3 antibody. The levels of β-actin or GAPDH were utilized as the loading control.

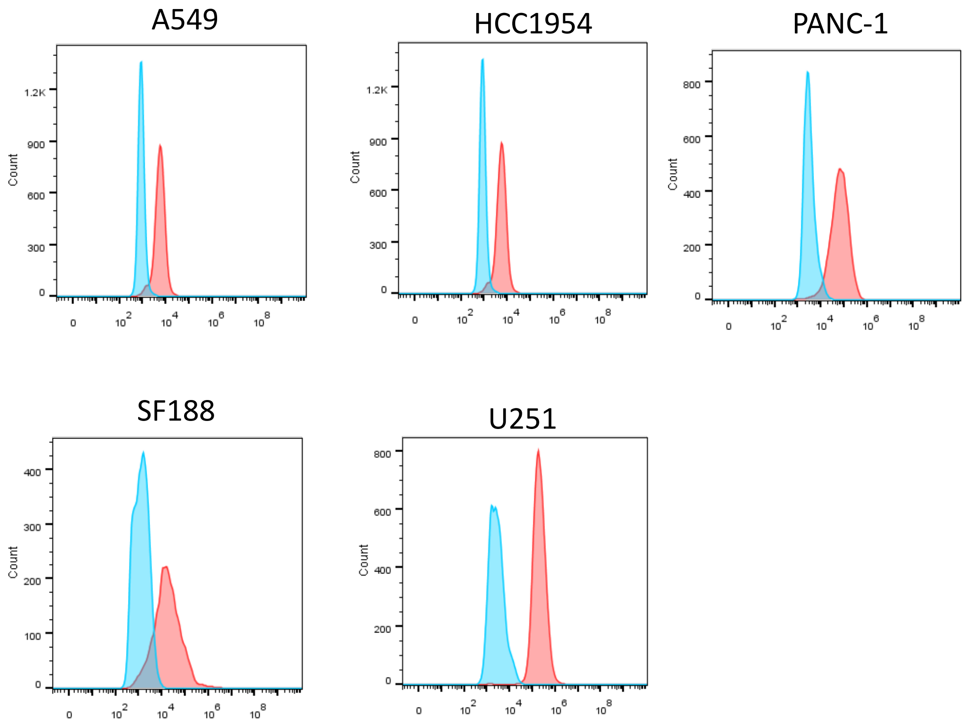

Figure S2. Expression of PD-L1 in five human tumor cell lines. Flow cytometric analysis of B7-H3 expression on the surface of tumor cells line. Immunostaining was was performed using PE conjugated anti-PD-L1 antibody (BioLegend). Red color represents PD-L1 staining. Blue color represents control antibody staining.

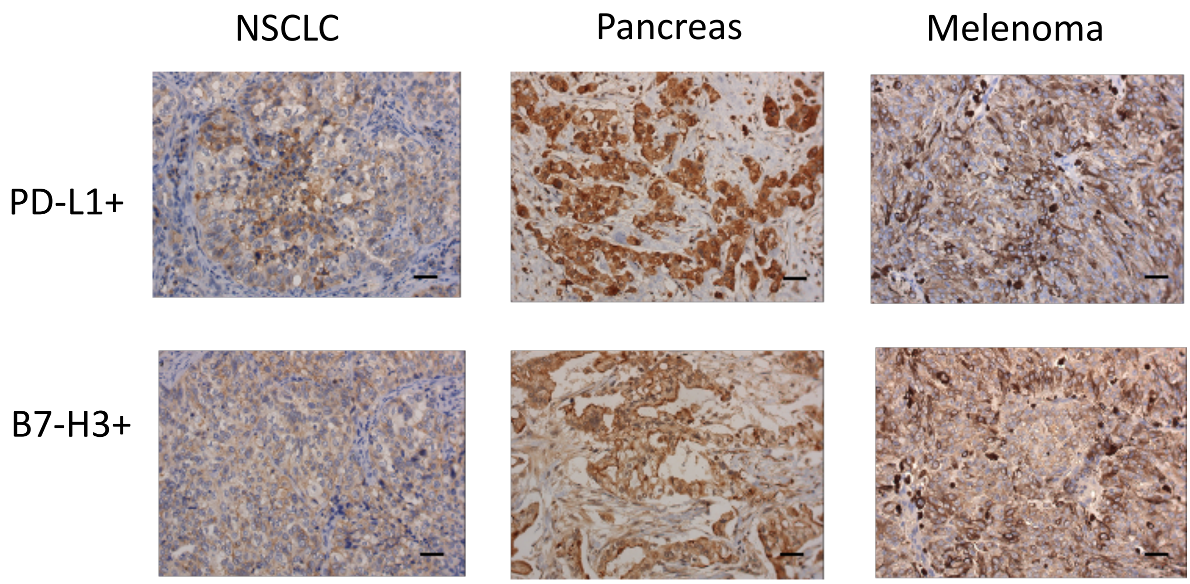

Tumor type	Number (B7-H3+)	Number (PD-L1+)
NSCLC	11	3
Pancreas	1	1
Melenoma	2	1

Figure S3. IHC staining of B7-H3+ human tumor tissues by anti-PD-L1 antibodies. The sale bar represents 20 μm.

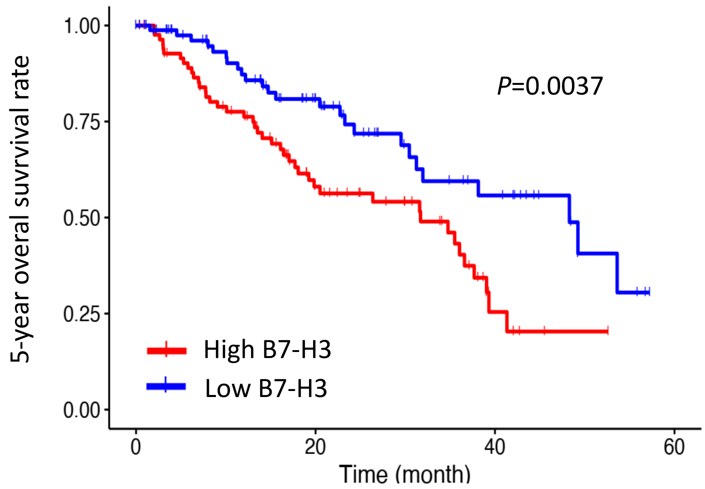

Figure S4. Relationship between B7-H3 expression and prognosis of patients with LUAD. Kaplane-Meier analysis of the 5-year overall survival rate according to high and low tumor B7-H3 levels (highest 15% B7-H3, *n*=108; lowest 85% B7-H3, *n*=108). The significance in survival differences was analyzed by the log-rank test.

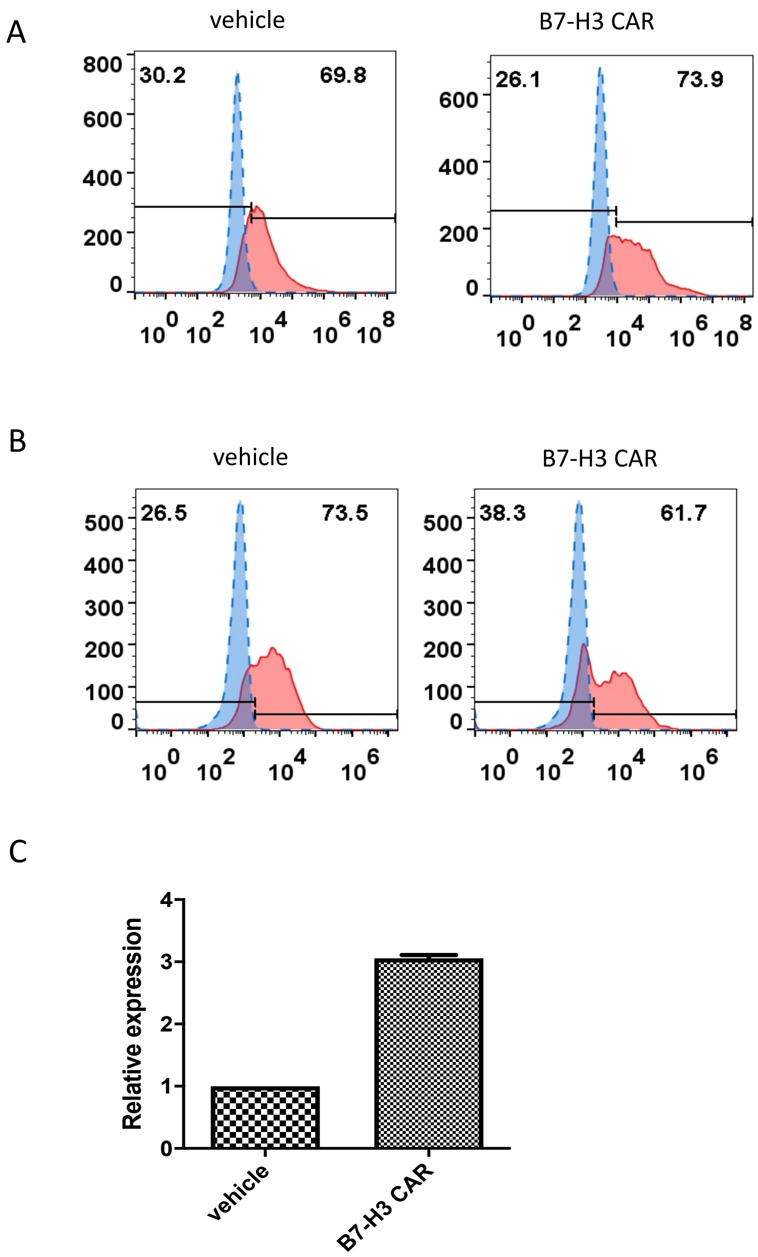

Figure S5. Expression of B7-H3 CAR in host cells. (A-B) Expression of B7-H3 CAR in 293T cells (A) and primary human T cells (B) after lentiviral transduction as assessed by ZsGreen fluorescence expression with flow cytometry. (C) The relative mRNA level of the B7-H3 CAR in primary T cells compared to vehicle T cells as analyzed by qPCR.

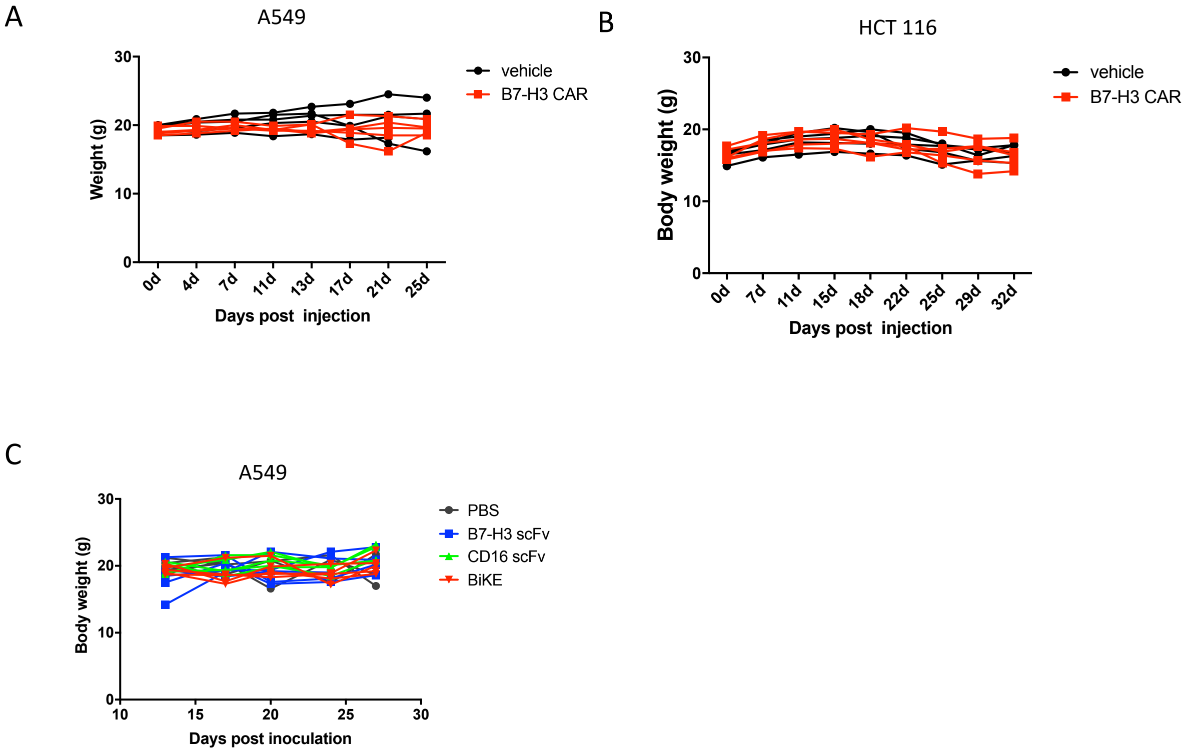

Figure S6. Body weight of tumor xenograted mice with treatment. (A, B) Curves of mouse body weight in A549-bearing mice (A) and HCT 116-bearing mice (B) treated by B7-H3 CAR T cells and vehicle T cells. (C) Body weight in A549-bearing mice treated by the B7-H3/CD16 BiKE, B7-H3 scFv, CD16 scFv and PBS.

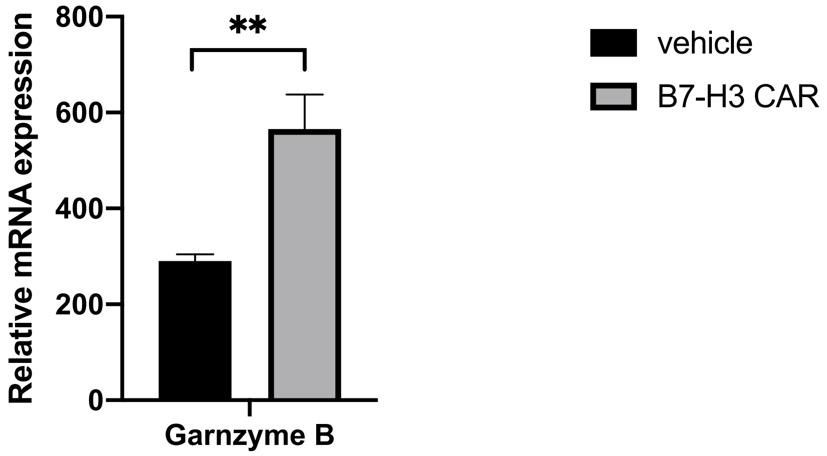

Figure S7. The relative mRNA level of granzyme B in B7-H3 CAR T cells compared to vehicle T cells. Effector T cells were incubated with target A549 cells at a ratio of 10:1 for 24 h. The mRNA levels of granzyme B were analyzed by qPCR. Data from 2 independent experiments are presented as the mean ± SD.

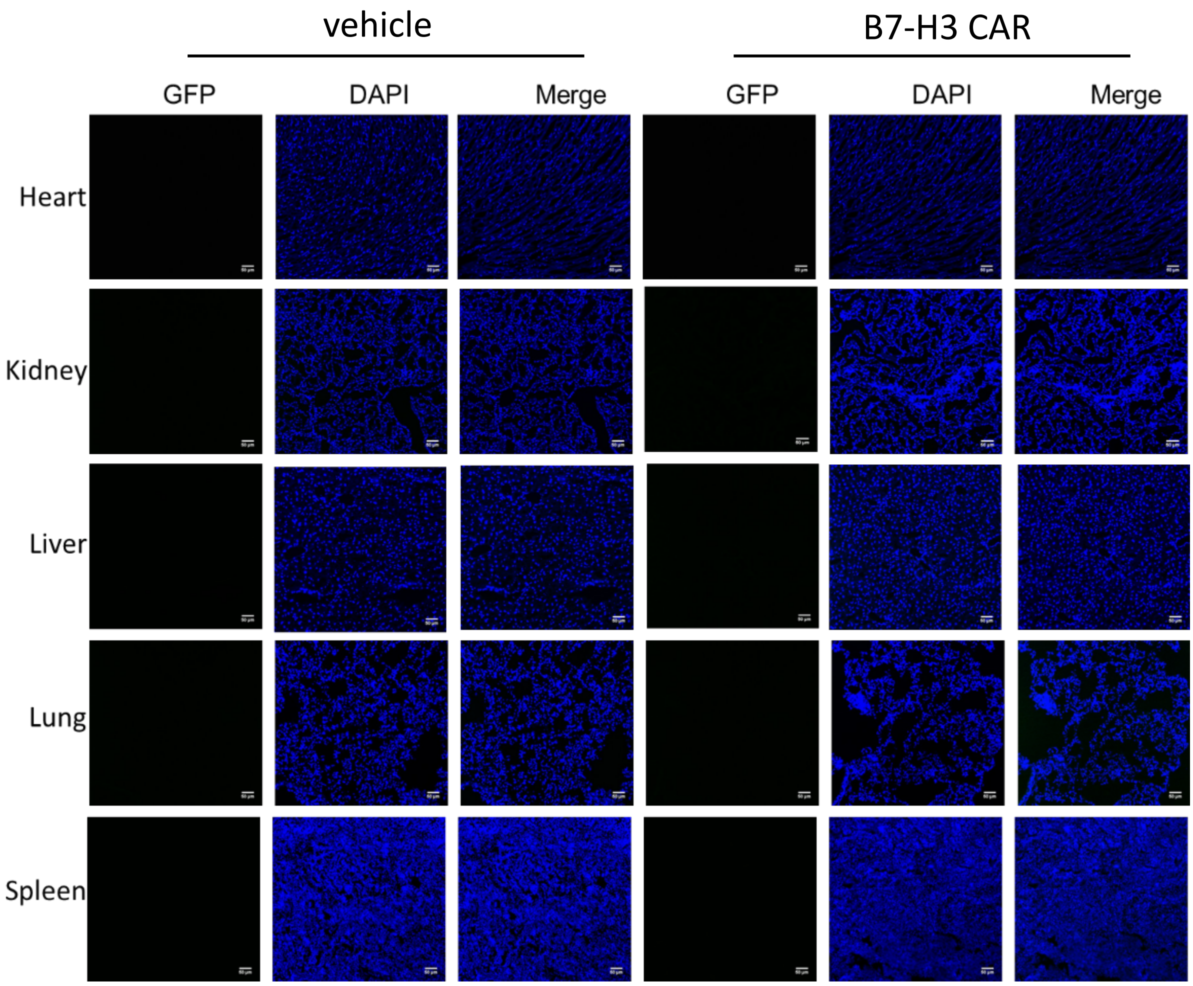

Figure S8. Biodistribution analysis of B7-H3 CAR T or vehicle T cells in major organs of treated mice. On day 2, organs were harvested, sliced, fixed, and imaged by the fluorescent microscope.

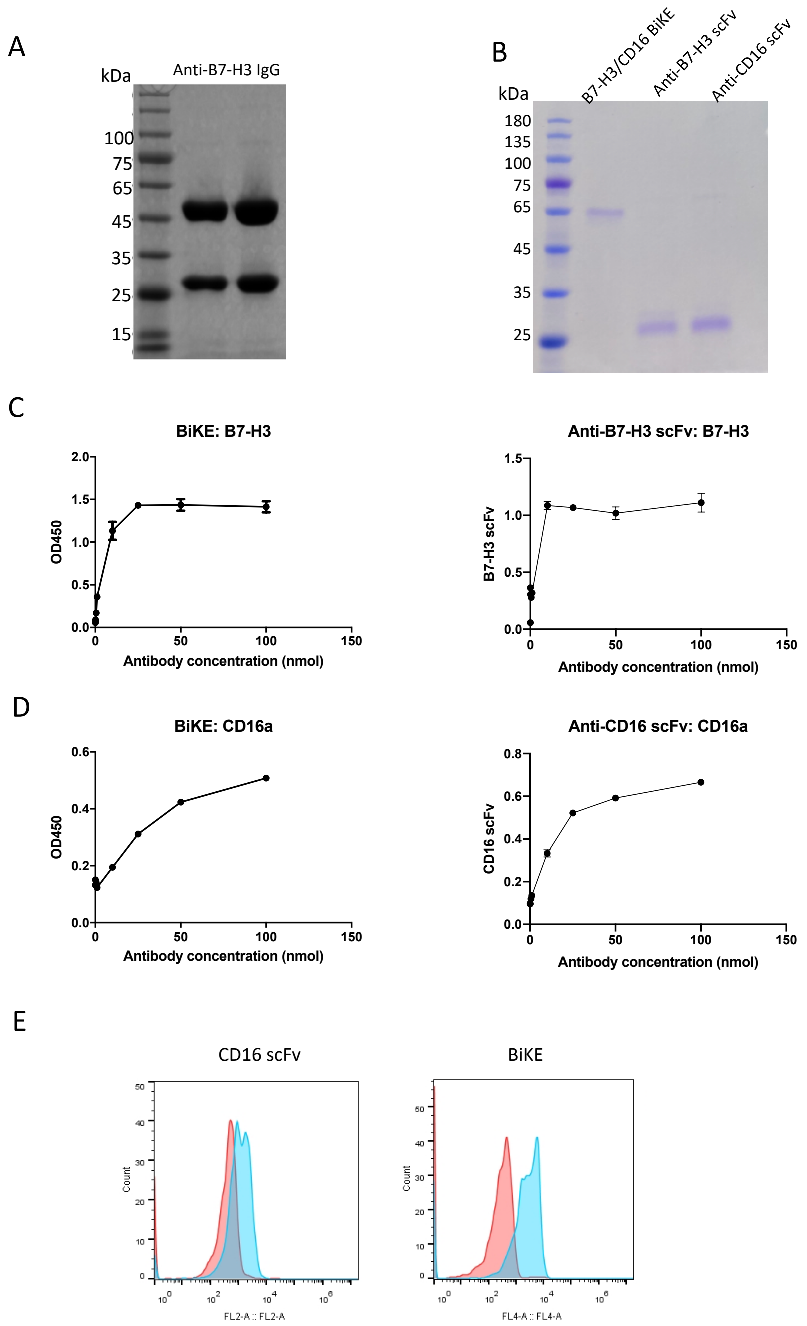

Figure S9. Production and characterization of anti-B7-H3 and anti-CD16 antibodies. (A-B) SDS-PAGE analysis of purified anti-B7-H3 IgG 8H9, B7-H3/CD16 BiKE, anti-B7-H3 scFv, and anti-CD16 scFv. (C) Binding of B7-H3/CD16 BiKE, anti-B7-H3 8H9 scFv to B7-H3 protein by ELISA. The anti-B7-H3 IgG 8H9 is used the positive control. (D) Binding of B7-H3/CD16 BiKE, anti-CD16 scFv to CD16a protein by ELISA. BiKE was detected by HRP-anti-c-myc antibody. The scFvs were detected by HRP-anti-FLAG antibody. One mouse anti-CD16 antibody 3G8 (BD Biosciences) is used as the positive control. (E) Binding of B7-H3/CD16 BiKE, anti-B7-H3 scFv to CD16a-expressing NK-92 cells by flow cytometry. The BiKE was detected by mouse anti-c-myc antibody, followed by APC-goat anti-mouse IgG. The anti-CD16 scFv was biotinylated and detected by PE-streptavidin. One irrelevant anti-HIV envelope scFv is used as the isotype control.

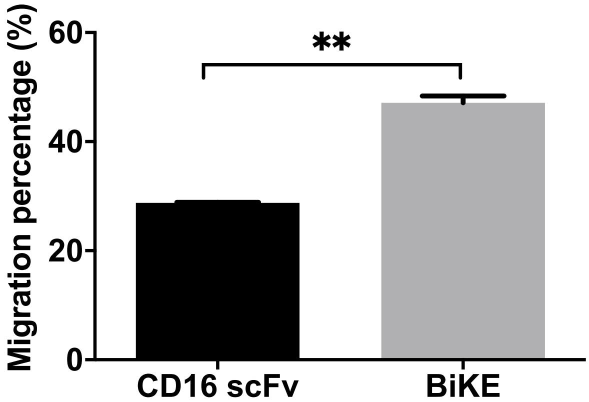

Fig.S10. NK cell migration induced by the B7-H3/CD16 BiKEafter and anti-CD16 scFv.

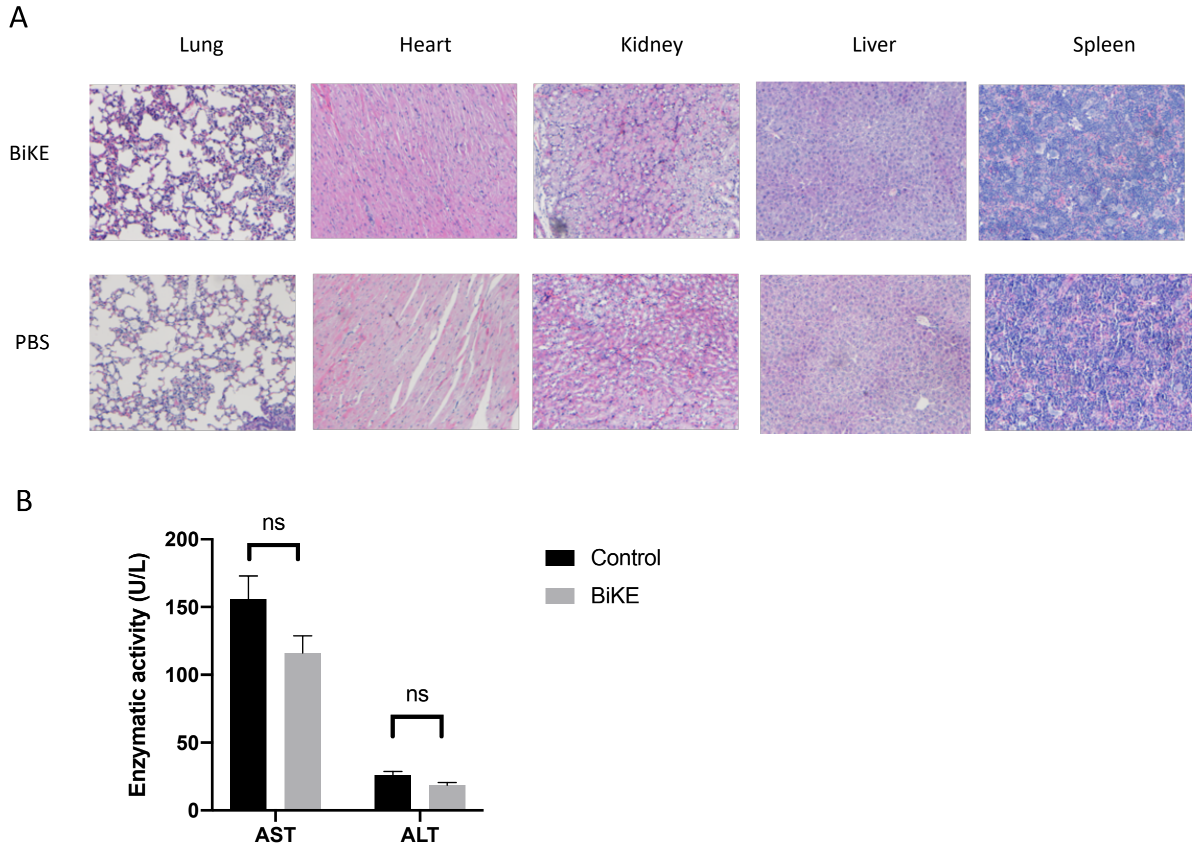

Figure S11. Toxicity evaluation of the B7-H3 x CD16 BiKE. (A) H&E staining images of major organs including Lung, heart, kidney, liver, and spleen after the mice were sacrificed post intravenous injection with BiKE. Bar represents 50 µm. (B) Serum levels of AST and ALT were measured for the BiKE and negative control groups.

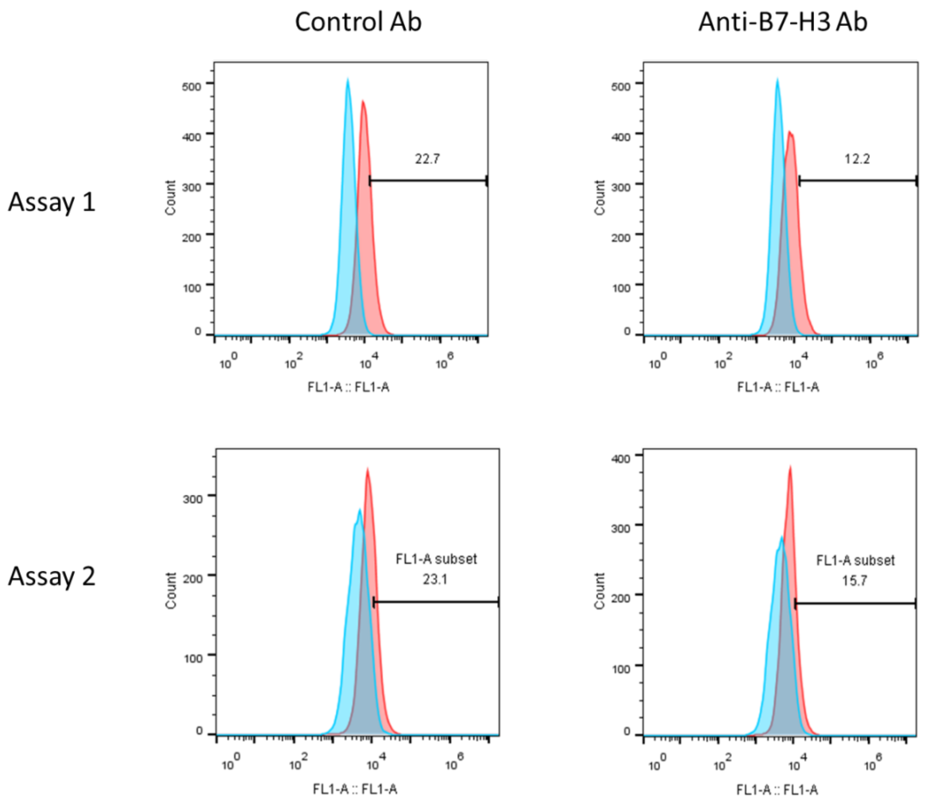

Figure S12. Intracellular ROS measurement in tumor cells. A549 cells were labeled with 2, 7-dichlorofluorescin diacetate as a probe and treated with the anti-B7-H3 antibody ot the control antibody. Intracellular ROS signals were measured by flow cytometry. The experiments were performed in two individual samples.

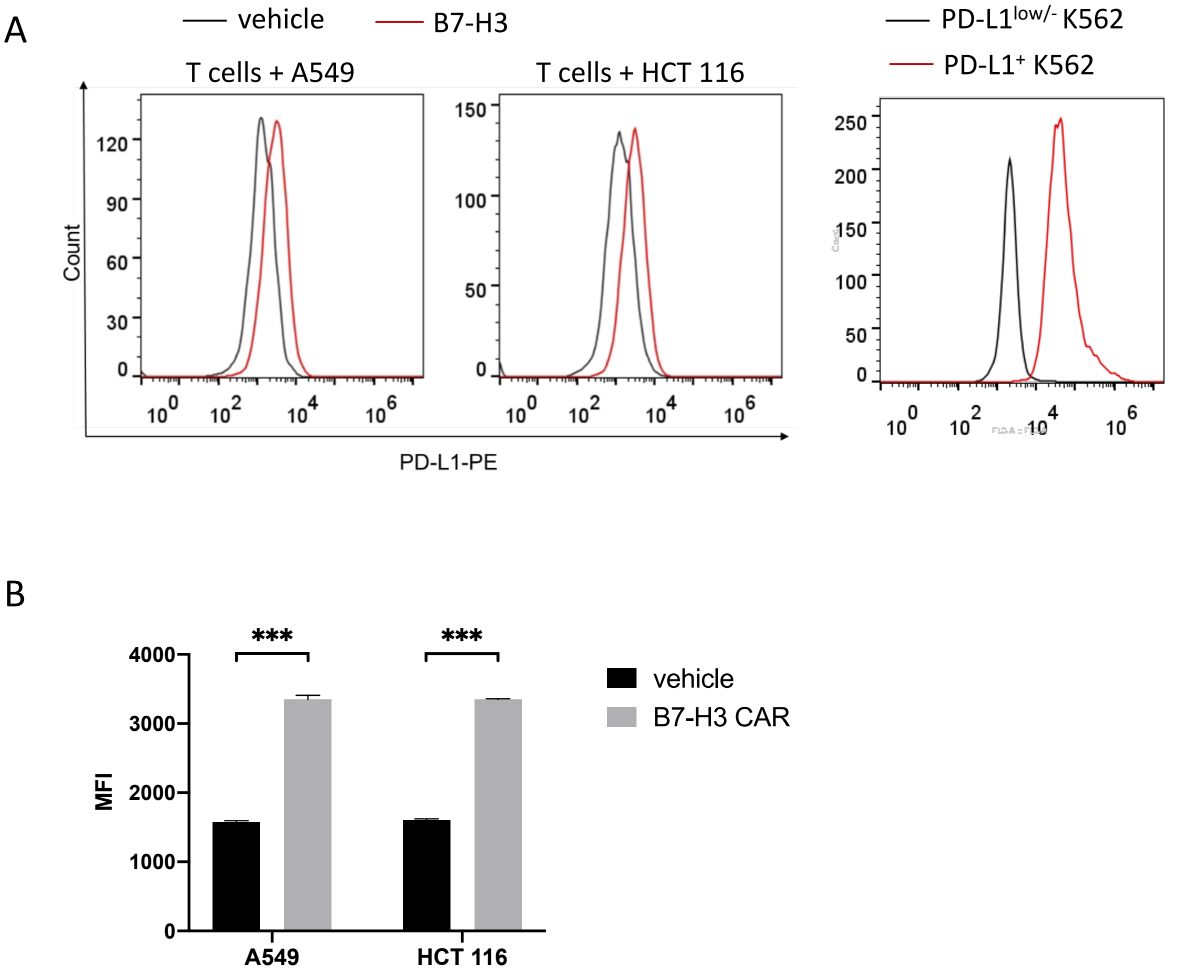

Figure S13. PD-L1 expression on the B7-H3 CAR T cells co-cultured with target cells. (A) Flow cytometry analysis was performed to analyze the PD-L1 expression on B7-H3 CAR and vehicle T cells after they were co-incubated with A549 and HCT 116 cells for 24 h, respectively. CD3+ T cells are gated by APC-conjugated anti-human CD3 antibody (300312, BioLegend). The expression of PD-L1 on the surface of cells are detected by the PE-conjugated anti-human PD-L1 antibody (329705, BioLegend). A human PD-L1 transfected K562 cell line is used the positive control of PD-L1. Representative results are shown for one experiment with two PBMC donors from three independent experiments. (B) Summary of PD-L1 expression represented as MFI in different effector T cells. The results are presented as the Mean ± SEM of three independent experiments.

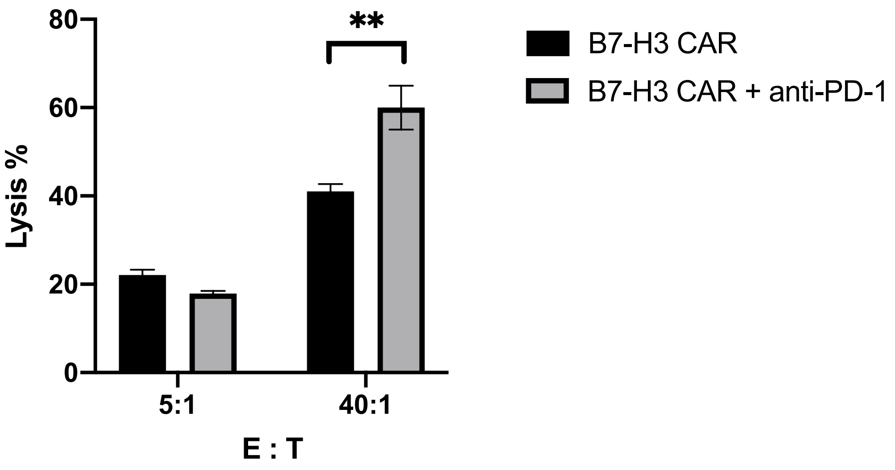

Figure S14. Cytotoxicity of B7-H3 CAR-T cells in combination with one anti-PD-1 antibody against A549 cells. Targeted cells were incubated with 10 μM of Calcein-AM for 30 min at 37 °C. After washing, targeted cells seeded at 1 × 10^4 cells/well in the 96-well plate were co-incubated with effector CAR-T cells with 10ug/ml of anti-PD-L1 antibody at different effector-to-target (E: T) ratios from 5:1 to 40:1 in a total volume of 200 μL for 4 h. Mean fluorescence intensity (MFI) in the supernatant was measured at 495/515 nm.

Table S1. Affinity measurement of BiKE and 8H9 scFv against 4Ig-B7-H3.

	Kon (1/Ms)	Kdis (1/s)	KD (M)
BiKE	5.42E+04	5.42E+04	2.94E-09
scFv	1.23E+04	7.96E-05	6.71E-09

The average K_D values were calculated based the results from two individual experiments.
